# A Comparison between Mouse, *In Silico*, and Robot Odor Plume Navigation Reveals Advantages of Mouse Odor Tracking

**DOI:** 10.1523/ENEURO.0212-19.2019

**Published:** 2020-01-31

**Authors:** A. Gumaste, G. Coronas-Samano, J. Hengenius, R. Axman, E. G. Connor, K. L. Baker, B. Ermentrout, J. P. Crimaldi, J. V. Verhagen

**Affiliations:** 1 Interdepartmental Neuroscience Program, Yale University, New Haven, CT 06510; 2 The John B. Pierce Laboratory, New Haven, CT 06519; 3 Department of Neuroscience, Yale School of Medicine, New Haven, CT 06510; 4 Department of Mathematics, University of Pittsburgh, Pittsburgh, PA 15260; 5 Department of Civil, Environmental and Architectural Engineering, University of Colorado, Boulder, CO 80309

**Keywords:** *in silico*, mouse, navigation, odor plume, robot, turbulence

## Abstract

Localization of odors is essential to animal survival, and thus animals are adept at odor navigation. In natural conditions animals encounter odor sources in which odor is carried by air flow varying in complexity. We sought to identify potential minimalist strategies that can effectively be used for odor-based navigation and asses their performance in an increasingly chaotic environment. To do so, we compared mouse, *in silico* model, and Arduino-based robot odor-localization behavior in a standardized odor landscape. Mouse performance remains robust in the presence of increased complexity, showing a shift in strategy towards faster movement with increased environmental complexity. Implementing simple binaral and temporal models of tropotaxis and klinotaxis, an *in silico* model and Arduino robot, in the same environment as the mice, are equally successful in locating the odor source within a plume of low complexity. However, performance of these algorithms significantly drops when the chaotic nature of the plume is increased. Additionally, both algorithm-driven systems show more successful performance when using a strictly binaral model at a larger sensor separation distance and more successful performance when using a temporal and binaral model when using a smaller sensor separation distance. This suggests that with an increasingly chaotic odor environment, mice rely on complex strategies that allow for robust odor localization that cannot be resolved by minimal algorithms that display robust performance at low levels of complexity. Thus, highlighting that an animal’s ability to modulate behavior with environmental complexity is beneficial for odor localization.

## Abstract (precis)

Localization of odors is essential to animal survival, and thus animals are adept at odor navigation. In natural conditions animals encounter odor sources in which odor is carried by air flow varying in complexity. We sought to identify potential minimalist strategies that can effectively be used for odor-based navigation and asses their performance in an increasingly chaotic environment.

## Significance Statement

A promising body of work has been devoted to designing robots and algorithms that address the strategies used by animals during odor-based navigation. One method to do so is by designing models that account for complex navigational tactics implemented by a particular species. How do these models directly compare to animal behavior in the same environment? We addressed this question by comparing odor-localization performance of minimal spatial and temporal algorithms *in silico* and in a robot to the strategies and performance of mice in the same odor environment. Through implementing this unique comparison, we revealed that mouse behavior remains robust with an increase in odor plume complexity, whereas simple algorithm behavior (although high performing at low plume complexity) does not.

## Introduction

Odor-based navigation is critical to animal survival as animals depend on olfactory cues to locate food sources, find mates, and avoid predators. Odors in nature are often carried by chaotic air or water flow, producing plumes with complex spatiotemporal structure. In large naturalistic environments, odor plumes become characterized by odor fluctuations, providing animals with a dynamic odor environment to navigate (Crimaldi et al., 2002; Connor et al., 2018).

Animals display a variety of behavioral strategies when navigating odor landscapes. Mammals exhibit zig-zagging casting behavior when tracking odor trails (Porter et al., 2007; Khan et al., 2012; Jones and Urban, 2018; Liu et al., 2019) and similarly, insects display casting behavior when traveling through airborne odor plumes (Willis and Avondet, 2005; Gomez-Marin et al., 2011). For both insects and crustaceans, odor plume complexity can affect odor-source localization (Mafra-Neto and Cardé, 1994; Keller and Weissburg, 2004). Moths exhibit a decrease in casting behavior and increase in fast, straight upwind paths in the presence of increased complexity, suggesting that complexity can be beneficial for odor tracking in some species. Although insect and crustacean behavior within odor landscapes has been studied for decades, a small but growing body of literature is focusing on the behavioral strategies used by mammals, specifically rodents, for airborne odor source localization. When rodents are tested on odor source localization in small flow-chambers where odor is released from a set of predictable locations, they ultimately predominantly use a habitual strategy relying on spatial memory to find odor ports (Bhattacharyya and Bhalla, 2015; Gire et al., 2016). Additionally, these studies suggest that rodents do not exhibit casting behavior during odor localization within airborne plumes, an interesting contrast to the casting observed during trail following.

To systematically determine the strategies that may account for animal odor-based navigation, scientists have turned to robotics. Several robotics-based approaches to odor localization have focused on replicating well-studied moth navigational strategies. These studies employed algorithms combining odor and wind-sensing to mimic casting behavior (Ishida et al., 1996; Harvey et al., 2008; Lochmatter et al., 2008; Lochmatter and Martinoli, 2009). Successful robotics strategies have implemented fans to actively draw air into sensors, similar to the beating of a moth’s wings, showing that fanning action causes a greater difference in perceived concentration between two sensors (Nakamoto et al., 1996). Although implementing robotic algorithms inspired by animal trajectories is useful when developing robust odor-source localization strategies, it is critical that the efficacy of these algorithms is tested through direct comparison with animals. Studies aimed at bridging the gap between simulations and real animal behavior have used insect antennas to replace sensors as well as used a robot to generate lobster antenna movements to study the resulting changes to the odor environment (Kuwana and Shimoyama, 1998; Koehl et al., 2001). Stereo smell is beneficial for odor localization in invertebrates and mammals alike (Porter et al., 2007; Catania, 2013; Jones and Urban, 2018). With unilateral naris occlusion, mouse odor localization accuracy drops and when input to one antenna is blocked, *Drosophila* fail to orient toward airborne odor plumes (Rajan et al., 2006; Duistermars et al., 2009). Thus, when developing algorithms to compare to animal odor-navigation behavior, it is essential to consider stereo smell. When tested in identical physical conditions to the milieu of a lobster, a RoboLobster implementing minimal algorithms based on a difference in concentration between two chemical sensors, displays paths that are both more tortuous and less successful when compared to an actual lobster (Grasso et al., 2000). This suggests that lobster odor-navigation strategy is more complex than a simple comparison between concentrations at two sensors.

Here, we directly compare the behavior of mice, minimal *in silico* odor-localization models, and an Arduino robot implementing these models (tropotaxis and klinotaxis) in the presence of two levels of odor plume complexity. The use of *in silico* models allows for flexibility of testing a variety of navigation strategies, supports the quantification of effects of varied sensor parameters and enables the measurement of instantaneous concentration during odor navigation. To the best of our knowledge, our study is the first to directly test airborne odor-navigation algorithms, designed *in silico*, implemented in a robot and real rodent behavior within the same flow chamber. We find that mouse odor localization remains robust in a plume which is increasingly chaotic, and that complexity may benefit the efficiency of navigation. Additionally, we find that when tested in the same environment as the mouse, an Arduino robot shows decreased performance with increased odor plume complexity, highlighting the robustness of mouse navigation behavior.

## Materials and Methods

### Standard odor landscape (SOL)

**Figure 1.**
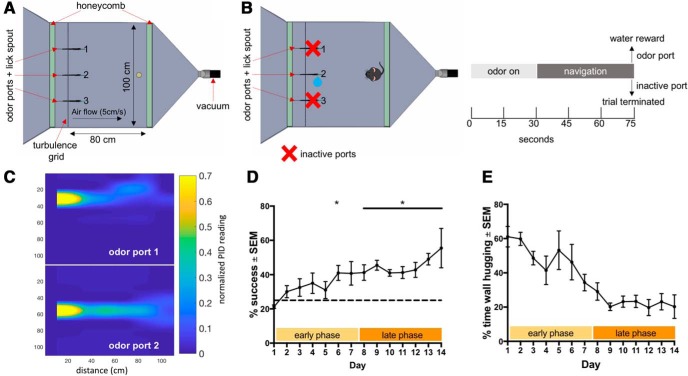
Mouse odor-navigation task. ***A***, Flow chamber used to conduct behavioral assay. Chamber is flanked by two honeycombs and on the inlet side, a turbulence grid 10 cm in front of the honeycomb. Three odor ports and lick spouts are spaced along inlet side and vacuum is used to establish air flow (5 cm/s). ***B***, Mouse is rewarded for navigating to the port releasing odor (port two) and trial is terminated early if animal navigates to incorrect port (left). Trial structure includes a 30-s period to establish plume before animal enters chamber and given 45 s to navigate (right). ***C***, miniPID readings of odor concentration from odor ports 1 and 2 (time averaged and normalized to maximum reading which occurs at the odor source). ***D***, Performance (% successful trials in a given session) of mice over testing days. Performance is broken up into an early phase (first 7 d) and a late phase (last 7 d). Plot shows mean performance ± SEM, *n* = 4 mice. ***E***, Percentage of time spent hugging the chamber wall, defined as within 5 cm of behavioral arena wall, over testing days. Plot shows mean % time spent wall hugging ± SEM, *n* = 4 mice. See also Extended Data Figure 1-1. *p < 0.05.

A SOL arena was built as described in Connor et al. (2018), barring a few adjustments related to the behavioral assay. The core of the flow chamber had dimensions of 100 cm wide, 100 cm long (in flow direction), and 30 cm tall. The chamber was flanked by honeycomb flow-straighteners (Plascore PC2-125-W-2 polycarbonate 1/8-inch cell, 2 inches thick, 1 × 0.3 m) and the air inlet had a turbulence grid (2.5 × 2.5 cm spacing, steel grid wire 3-mm OD) 20 cm downstream of the inlet honeycomb (Fig. 1*A*). Airflow of 5 cm/s was established using a vacuum attached to the outlet of the flow chamber. The inlet side of the flow chamber tapered from a surface area of 1.2 m^2^ to the 0.3 m^2^ of the main arena (where the inlet honeycomb was placed). Isoamyl acetate (IAA; 3% in mineral oil, Sigma-Aldrich) was released, also at 5 cm/s, through one of three odor tubes magnetically clipped on to and extending 10 cm in front of the turbulence grid. Each odor tube was an 18-cm-long 3-D printed horn linearly expanding from an inner diameter of 3–10 mm and its lower edge raised 15 mm above the floor (horn center at 20 mm off the floor). Odor tubes were located at midline and 25 cm lateral to midline. An air-dilution olfactometer was built to deliver odor by bubbling air through an odor vial containing 3% IAA in mineral oil. Each odor tube isokinetically delivered either air or odor at 236 ml/min. Above each odor port was a lick spout associated with that port. In the case of robot testing, LED lights were attached on top of each odor port in place of the lick spouts. All sides of the flow chamber were constructed from white acrylic and the top of the flow chamber was constructed from clear acrylic to allow for imaging during the behavioral task. A 2-inch diameter hole was cut in the base of the flow chamber directly in front of the outlet honeycomb (center at 7.5 cm) along the midline (from downstream to upstream) of the chamber. This hole served as the insertion point for animals at the beginning of every trial and was immediately sealed after animal entry using a magnetic disk that was flush with the base of the flow chamber.

10.1523/ENEURO.0212-19.2019.f1-1 Extended Data Figure 1-1 Odor plume within the SOL with and without honeycomb. ***A***, Odor plume properties within the SOL with and without the inlet air laminarization honeycomb at 10, 30, 50, and 60 cm downstream from odor tube. The average miniPID reading at 50 cm from the odor tube is greater without the honeycomb when compared to with the honeycomb (one-tailed *t* test with correction for multiple comparisons, average with honeycomb 0.16 ± 0.04, average no honeycomb: 0.36 ± 0.06, *p* = 0.040). The SD of the PID reading at all distances from the outlet is greater without the honeycomb than with the honeycomb (one-tailed *t* test with correction for multiple comparisons, 60-cm std with honeycomb: 0.09 ± 0.02, 60-cm std no honeycomb: 0.18 ± 0.02, *p* = 0.014; 50-cm std with honeycomb: 0.10 ± 0.02, 50-cm std no honeycomb: 0.18 ± 0.01, *p* = 0.004; 30-cm std with honeycomb: 0.11 ± 0.01, 30-cm no honeycomb: 0.25 ± 0.02, *p* < 0.0001; 10-cm with honeycomb: 0.08 ± 0.03, 10-cm no honeycomb: 0.32 ± 0.01, *p* < 0.0001). The std/average is greater without the honeycomb than with the honeycomb at 30 and 10 cm from the odor tube (one-tailed *t* test with correction for multiple comparisons, 30-cm std/average with honeycomb: 0.29 ± 0.04, 30-cm std/average no honeycomb: 0.57 ± 0.09, *p* = 0.033; 10-cm std/average with honeycomb: 0.08 ± 0.02, 10-cm std/average no honeycomb: 0.32 ± 0.04, *p* < 0.0001). ***B***, Example PID readings for honeycomb and no honeycomb conditions from 2-min sample at 60 cm from the source. ***C***, Example PID readings for honeycomb and no honeycomb conditions from 2-min sample at 30 cm from the source. Download Figure 1-1, TIF file.

To increase lateral variation in the flow which in turn increases the chaotic mixing (Mehta and Bradshaw, 1979) in the SOL, we removed the inlet honeycomb, allowing ambient room air flow to add complexity in addition to the static turbulence grid (Fig. 1*A*; Extended Data Fig 1-1). To evaluate the effectiveness thereof, we measured odor concentration time series along the midline of the SOL at 10, 30, 50, and 60 cm downstream from the odor tube. Three series of 120 s (50 samples/s) were taken at each location with the inlet honeycomb, after which the honeycomb was removed and the measurements were repeated. This entire sequence was repeated once for a total of six-time series per location per condition (Extended Data Fig. 1-1). Measurements were taken with a miniPID (Aurora Scientific) set to low gain and slow pump speed. The odor used was 50% ethanol evaporated via a stainless-steel bubbler and released isokinetically (flow conditions were identical to the experimental conditions described above). To minimally interfere with the non-turbulent chaotic airflow and ensure measurement consistency, the midline and upstream edges of the miniPID sensor body were located 15 cm lateral from midline and 5 cm downstream from the inlet tip of a 1/8-inch OD Teflon tube bent gradually at 90° to suck in air in downstream direction. A 22-gauge needle pierced the tube vertically, 2 cm from the tube’s tip, and assured a consistent sampling height of 20 mm. The miniPID output was directly digitized using a Syscomp 11-bit A/D board (CGM-101) and streamed to disk. The final 6000 samples of each data file were saved as MATLAB data files (https://doi.org/10.5061/dryad.zgmsbcc71) and used for analysis of complexity (MATLAB code file, available on DataDryad, https://doi.org/10.5061/dryad.zgmsbcc71). Small DC-offsets were removed. Intermittency was calculated as the fraction of time the time series was above 4.4% of the maximum average signal at 10 cm from the odor tube.

### Mouse: behavioral training

Four adult male C57Bl/6 mice aged 24–26 weeks were used. Mice were handled for 20 min each day for one week before habituation in the flow chamber. Following a week of handling with the experimenter, animals were allowed to explore the flow chamber for 30 min/d for 5 d. Subsequently, animals were water regulated (body weight closely monitored and maintained at 85% of original weight) and trained to associate the lick spouts with sucrose water (100 mM) delivery. Water was dispensed free-flowing from each of the three lick spouts and animals were lick-trained until they licked from all three lick-spouts. Once lick-trained, animals were trained on a simple version of the navigation task. At the beginning of every trial, an odor plume was established from odor port 1 for 30 s, and then the animal was inserted into the arena through the 2-inch hole at the outlet end of the flow chamber. Animals were given 45 s to navigate to port 1 and were trained on this task for 6 d. Animals were group housed in an environment of controlled humidity (40%) and temperature (22°C) with a 12/12 h inverted dark/light cycle with lights off at 9 A.M. Animals were tested during their dark cycle under red light. All experimental protocols were performed in accordance with protocols approved by Pierce Animal Care and Use Committee. The John B Pierce Laboratory is AAALAC accredited. These procedures are in agreement with the National Institutes of Health Guide for the Care and Use of Laboratory Animals (8th edition).

### Mouse: odor navigation task

On each trial odor was released from one of three possible odor ports and isokinetic clean air was released from the other two ports. Thirty seconds was allotted for the odor plume to be established before inserting the animal. On entering the flow chamber through the 2-inch hole at the outlet end, the animal was given 45 s to navigate to the odor source. If the animal reached the correct odor source, an 8-kHz tone was played, and the animal was required to remain within the reward zone for 100 ms before a sucrose water reward (100 mM) was delivered for 500 ms. After sucrose water delivery, the animal was removed from the arena. If the animal approached an incorrect odor port or failed to reach the reward zone within the 45-s duration, a 1-kHz tone was played and the animal was removed from the arena. In between trials odor was turned off and the animal was placed in an enrichment cage for 45 s. This amount of time was sufficient to clear any residual odor from the flow chamber. Animals were tested on 30–40 randomized trials per day with equal representation of each odor port. Animals were tested using the honeycomb condition for 14 d and subsequently without the honeycomb for 5 d. Lastly, animals were tested on a no odor control paradigm.

### Model: geometry

We developed *in silico* simulations of odor navigation in static and dynamic plumes. We refer to these simulations interchangeably as a model and simulated robot. The simulated robot makes temporally discrete sample-to-sample comparisons of odor concentration at its left and right sensors as it moves through space. It consists of a virtual chassis with coordinates centered at (*x, y*) and moves through space along a heading *θ* at a velocity *v*:

(1) $$x_{t+\Delta t}=x_{t}+v\text{ Δ}t\cos\theta$$

(2) $${\text{y}}_{t+\Delta t}=y_{t}+v\text{ Δ}t\sin\theta ,$$

where Δ*t* represents the update rate of the model, here 100 ms. Velocity *v* is 4 cm/s. The agent has a chassis radius of *ℓ_d_* = 8 cm. Sensors are located at the front of the chassis with a variable intersensor distance of *ℓ_s_*. The two sensors are separated by an angle γ = arctan($\frac{\ell_{s}}{2\ell_{d}}$). Sensor positions are given as:

(3) $$x_{L/R}=x+\sqrt{\ell_{d}^{2}+{\left(\frac{\ell_{s}}{2}\right)}^{2}}\cos\left(\theta \pm \gamma \right)$$

(4) $$y_{L/R}=y+\sqrt{\ell_{d}^{2}+{\left(\frac{\ell_{s}}{2}\right)}^{2}}\sin\left(\theta \pm \gamma \right),$$

where (*x_L_*, *y_L_*) is the left sensor and (*x_R_*, *y_R_*) is the right sensor. The agent geometry is shown in Figure 3*A*.

The simulated robot engages in hierarchical navigation algorithms which begin with (1) baseline acquisition, followed by iterative (2) wall avoidance, and then (3) odor-driven navigation. Both baseline acquisition and odor-driven navigation require transduction of the underlying odorant concentration into a sensor signal.

### Model: odor signal simulation

Odor signals at each sensor are simulated as

(5) $${\dot{S}}_{L/R}=-k_{decay}S+C_{x,y,t}.$$

Here, k_decay_ is a rate constant set to ln(2)/0.8s on experimental sensor half-life data (Extended Data Fig. 4-1*C*). C_x,y,t_ represents the instantaneous concentration sampled at time t from the plume dataset at the pixel position (point source) corresponding to either the left or right sensor.

Using this simple model for sensor odor signal, we may define the model’s baseline acquisition and odor-driven navigation.

#### Baseline acquisition

Baseline acquisition is identical for both simulated algorithms. First, the simulated robot remains stationary for 10 s to allow its sensors to equilibrate according to Equation 5.

After equilibration, the model remains stationary and samples from the left sensor four times over the following second. These sensor values are averaged to generate *S_L,baseline_*. Over the subsequent second model performs the same procedure at the right sensor to generate *S_R,baseline_*.

Finally, the two baselines are averaged to obtain *S_baseline_* = (*S_L,baseline_* + *S_R,baseline_*)/2, a value which will be used in odor-driven navigation.

#### Wall avoidance

In each loop of the simulated robot program, the model first uses its IR sensors to determine whether it must take corrective action to avoid an arena wall. If the simulated robot’s center (*x*, *y*) approaches within distance *d_threshold_* = 10 cm of a wall, it takes the following corrective actions.

If the model approaches a wall from its left-hand side (i.e., if the wall is in the left IR detection radius in Fig. 3*A*, orange arc), it first turns right for 100 ms, corresponding to a change in heading of ∼30° to the right:

(6) $$\theta_{t+\Delta t}=\theta_{t}-\frac{\pi }{6}$$

It then moves forward for 200 ms according to Equations 1, 2.

If the model approaches a wall from its right-hand side (i.e., if the wall is in the right IR detection radius in Fig. 3*A*, green arc), it first turns left for 100 ms, corresponding to a change in heading of ∼30° to the left:

(7) $$\theta_{t+\Delta t}=\theta_{t}+\frac{\pi }{6}.$$

It then moves forward for 200 ms according to Equations 1, 2.

If the model approaches a wall head on (i.e., if the wall is in the center IR detection radius in Fig. 3*A*, blue arc), it first turns right for 100 ms, corresponding to a change in heading of ∼30° to the right (Eq. 6). It then backs up for 200 ms according to Equations 1, 2 (*v* = –4 cm/s to reverse course).

Following any of the above scenarios, the model remains stationary for 300 ms to allow the sensors to equilibrate.

### Model: odor-driven navigation

If no wall is encountered in a loop of the robot code, it engages in odor-driven navigation. Here, model behavior varies depending on whether algorithm A or B is implemented.

In algorithm A, the sensors are queried and one of three alternatives is selected based on current sensor values in order of precedence:

1. If the value (S_L_-S_baseline_) – (S_R_-S_baseline_) > S_threshold_, (S_threshold_ = 0.03), the model turns left for 100 ms according to Equation 7. It then moves forward for 200 ms according to Equations 1, 2.
2. If the value (S_R_-S_baseline_) – (S_L_-S_baseline_) > S_threshold_, the model turns right for 100 ms according to Equation 6. It then moves forward for 200 ms according to Equations 1, 2.
3. If neither 1 nor 2 occur, the model goes straight for 200 ms according to Equations 1, 2.

Following any of the above three scenarios, the model remains stationary for 300 ms to allow the sensors to equilibrate.

In algorithm B, memory of the previous average odor sample is retained. The sensors are queried and the temporal difference in average concentration values is computed:

(8) $$\Delta \bar{C}=\frac{1}{2}\left[{\left(\left(S_{L}-S_{threshold}\right)+\left(S_{R}-S_{threshold}\right)\right)}_{t}-{\left(\left(S_{L}-S_{threshold}\right)+\left(S_{R}-S_{threshold}\right)\right)}_{t-\Delta t}\right].$$

Using this value and the sensor values, one of four alternatives is selected based on current sensor values in order of precedence:

1. If $\Delta \bar{C}$> S_threshold_/4, the model goes straight for 200 ms according to Equations 1, 2.
2. If the value (S_L_-S_baseline_) – (S_R_-S_baseline_) > S_threshold_/2, the model turns left for 100 ms according to Equation 7. It then moves forward for 200 ms according to Equations 1, 2.
3. If the value (S_R_-S_baseline_) – (S_L_-S_baseline_) > S_threshold_/2, the model turns right for 100 ms according to Equation 6. It then moves forward for 200 ms according to Equations 1, 2.
4. If none of the above are true, the model proceeds forward for 200 ms according to Equations 1, 2.

Following any of the above three scenarios, the model remains stationary for 300 ms to allow the sensors to equilibrate. For algorithm A and B, the simulated robots are allotted 75 s to find the odor source.

### Model: plume data

Four minutes of near-surface acetone planar laser-induced fluorescence (PLIF) plume data from Connor et al. (2018) was used as input for these models (11282017_10cms_bounded.h5,/dataset7).The above models are deterministic. If they are synchronized with the first frame of the plume dataset, they will always generate the same trajectory. To simulate “random” complexity, each model evaluation initialized the plume dataset at a randomly chosen frame between 1 and 3600; the 4-min dataset was then allowed to loop continuously until the simulation concluded (Movie 1, Movie 2).

Movie 1. In silico dynamic plume released from corner port. Video played at 10 Hz (first 10 s shown). 10.1523/ENEURO.0212-19.2019.video.1

Movie 2. In silico dynamic plume released from center port. Video played at 10 Hz (first 10 s shown). 10.1523/ENEURO.0212-19.2019.video.2

To study the effect of a non-turbulent low chaos environment on model performance, we took the time average of the 4 min of plume data to generate a smooth static environment (Fig. 3*C*).

### Robot: design

We purchased and modified an Arduino robot (Extended Data Fig. 4-1*A*; Arduino robot, code: A000078, Arduino was purchased from Robotshop). The Arduino robot comes equipped with a control board (on top) with a control pad to turn ON/OFF the robot, an LCD screen to read the sensory data, a compass, a processor, and analog/digital inputs to attach a variety of sensors. Additionally, the robot contains a motor board (on bottom) with two wheels for movement, a processor, ON/OFF switch, a power jack (9 V), an interboard connector, a reset button for troubleshooting and a USB port to connect the robot with any device or computer. The robot can be programmed using Arduino software (Arduino Software IDE, 1.8.5 version). The same bare robot platform was also used for gas source localization by Ali Yeon et al. (2018).

To power the hardware, we mounted three step-down buck DC-DC converters (DROK, 3A) connected to three dual lithium ion battery (Samsung 18650, 3.6 V, 3000 mAh) holders connected in parallel, providing 3.0 V (fans), 5.0 V (robot), and 6.5 V (gas sensors). Two (left and right) gas sensors [DFRobot, Analog Gas Sensor, MQ-2 (DFROBOT) an Arduino package based on MQ-2 gas sensor by the Hanwei Electronics Co. (hwsensor)] with a high sensitivity to detecting alcohol (and a variety of volatile organic compounds such as LPG, methane, hydrogen and smoke) were installed on the robot (Extended Data Fig. 4-1*A*). The gas sensor’s tin oxide layer on the aluminum oxide ceramic tube is heated by a nickel-chromium alloy coil and has an odor concentration-dependent resistance, suitable to detect a range of concentrations of gasses at constant temperature and humidity. To increase the response speed (Extended Data Fig. 4-1*C*), both gas sensors were modified by drilling a hole in the PC-board behind the sensor and attaching a gas sensor fan (10 × 10 × 5 mm, UF3A5-100, Sunon, run at 3.0 V, 0.9 l/min) to suck in air from the front to back, and removing the front of the perforated metal grid. The sensors were powered at 6.5 V instead of the standard 5 V. Also, we designed a pair of 3-D printed holders, rods and clamps, to incorporate the gas sensors at the top of the robot to allow adjustment of the distance between them and their angle in the horizontal plane. In addition, we added an analog ambient light sensor (DFRobot, V2, SKU:DFR0026) mounted at the front of the robot at the base of a frontally oriented cone and three IR-based proximity sensors (Sharp, GP2Y0A41SK0F; Extended Data Fig. 4-1*A*) at the center, left and right sides on the top board. Codes run on the Arduino robot are in accordance with the algorithm A and B described for the *in silico* model. These algorithms have been made available DataDryad (https://doi.org/10.5061/dryad.zgmsbcc71).

Response dynamics of the gas sensors were evaluated with a custom Arduino code reading the sensor voltage 100 times per second. Sensors were stimulated by rapidly manually passing an alcohol-saturated cotton swab from left to right at 0.5 inches from the frontal plane of the sensors ∼15.2 s after starting a trial. The baseline reading (mean of first 100 samples) was subtracted and response maxima were normalized to 1. Individual responses were time-aligned to the peak and smoothed with a three-sample running average. Reported data are averages of 2–10 trials, ignoring several trials with more than one peak and/or non-exponential decay.

### Robot: odor navigation task

As in the mouse odor navigation task, at the beginning of every trial, odor was released from one of three odor ports and isokinetic air flow was released from remaining two ports. Odor plume was established for 10 s before the beginning of the trial. The real robot, as opposed to the simulated *in silico* robot described above, was allotted 75 s to navigate to the odor source. The robot was tested on odor navigation to all three odor ports from varying start angles from a center start position along the midline of the outlet end of the flow chamber. For odor port 1 (right-most odor port), the robot was tested at start angles of 90°, 135°, and 180°, for odor port 2 (center odor port), the robot was tested at start angles of 135°, 180°, and 225°, for odor port 3 (left-most odor port) the robot was tested at start angles of 180°, 225°, and 270° (Fig. 4*A*). For each of these start angles, the robot was tested once with sensor angles of 0^°^ and 45^°^ as well as with sensor distances of 8 and 16 cm. Both Code A and Code B were tested in the presence of the honeycomb and Code B was tested without the honeycomb. The robot was also tested from a corner start position where it was located at a 270° angle at the right-most corner of the outlet end of the flow chamber. This start position was tested using active odor port 2. For this start position the robot was tested once with sensor angles of 0° and 45° as well as with sensor distances of 8 and 16 cm. Both Code A and Code B were tested with and without the honeycomb for this start position. The robot was tested for 10 trials for every condition.

### Code accessibility

All codes have been made available on DataDryad (https://doi.org/10.5061/dryad.zgmsbcc71). Additionally, all codes are in Extended Data 1. Included are MATLAB and Arduino codes to generate the center and corner odor plumes (file names: odorFun_plume_center.m, odorFun_plume_corner.m), test the *in silico*-simulated robot using Code A and Code B (filenames: SimRobot_test_A.m, SimRobot_test_B.m), and to test the *in silico* model with replicates (filenames: run_model_A_replicates.m, run_model_B_replicates.m). Additionally, this folder contains two Arduino codes for robot navigation (file names: Robot_CodeA.ino, Robot_CodeB.ino). These files were run on Windows 10.

10.1523/ENEURO.0212-19.2019.ed1 Extended Data 1 In silico MATLAB and Arduino codes. Included are MATLAB codes to generate the center and corner odor plumes (file names: odorFun_plume_center.m, odorFun_plume_corner.m), test the *in silico*-simulated robot using Code A and Code B (filenames: SimRobot_test_A.m, SimRobot_test_B.m), and to test the *in silico* model with replicates (filenames: run_model_A_replicates.m, run_model_B_replicates.m). Additionally, the two Arduino codes for robot navigation (file names: Robot_CodeA.ino, Robot_CodeB.ino). Download Extended Data 1, ZIP file.

### Behavioral tracking and data analysis

All behavioral tracking, for both the mouse and robot, was conducted using Noldus behavioral tracking system (EthoVision XT, version 10.1, Noldus Information Technology) and trajectories were further analyzed using MATLAB (R2018a, The MathWorks). GraphPad Prism (version 7; GraphPad Software, Inc.) was used to generate graphs and conduct statistical analyses. For all group comparisons, statistical tests were corrected for multiple comparison using a Bonferroni correction when appropriate (Table 1). Mouse data represents the average for each mouse across all days for the given condition. Robot data represents the average across 10 trials per condition. Model data represents the average across 20 simulations. All data are represented as mean ± SEM.

**Table 1.** Statistical analyses

Location	Data structure	Statistical test	95% confidence Intervals
a	Paired % time spent wall-hugging (late phase vs early phase), *n* = 4 mice	Paired one-tailed *t* test	–35.91 to –18.15
b	Paired % success (late phase vs early phase), *n* = 4 mice	Paired one-tailed *t* test	–1.79 to –21.51
c	Paired % success (no honeycomb condition vs late phase), *n* = 4 mice	Paired two-tailed *t* test	–10.64 to 6.81
d	% success for honeycomb and no honeycomb conditions per odor port	Two-way ANOVA on % success (factors: port #, plume complexity)	Bonferroni correction: –3.8 to 56.2
e	% success for honeycomb and no honeycomb conditions per odor port	Two-way ANOVA on % success (factors: port #, plume complexity)	Bonferroni correction: –1.65 to 58.35
f	% success for honeycomb and no honeycomb conditions per odor port	Two-way ANOVA on % success (factors: port #, plume complexity)	Bonferroni correction: –27.85 to 32.15
g	Paired % success (no odor vs late phase), *n* = 4 mice	Paired one-tailed *t* test	–51.18 to –11.46
h	Paired % success (no odor vs no honeycomb condition), *n* = 4 mice	Paired one-tailed *t* test	–46.02 to –12.78
i	Paired distance to odor source on successful trials (late phase vs early phase)	Paired two-tailed *t* test	–114.2 to –7.34
j	Paired time to odor source on successful trials (late phase vs early phase)	Paired two-tailed *t* test	–6.92 to –2.28
k	Paired distance to odor source on successful trials (no honeycomb vs late phase)	Paired two-tailed *t* test	–25.94 to 18.91
l	Paired time to odor source on successful trials (no honeycomb vs late phase)	Paired two-tailed *t* test	–25.94 to 18.91
m	Paired average velocity during trial (no honeycomb vs late phase)	Paired two-tailed *t* test	0.49 to 15.59
n	Paired average angle sum during trial (no honeycomb vs late phase)	Paired two-tailed *t* test	–69.8 to 15.41
o	Paired average Δ nose angle (no honeycomb vs late phase)	Paired two-tailed *t* test	0.008 to 0.12
p	Average nose/body distance ratio (late phase)	One-sample two-tailed *t* test	1.13 to 1.15
q	Average nose/ body distance ratio (no honeycomb)	One-sample two-tailed *t* test	1.14 to 1.26
r	% success for static and dynamic across Code A and Code B, sensor distance 8 and 16 cm	Three-way ANOVA on % success (factors: plume complexity code, and sensor separation distance)	Bonferroni correction: 5.18 to 11.56
s	% success for static and dynamic across Code A and Code B, sensor distance 8 and 16 cm	Three-way ANOVA on % success (factors: plume complexity code, and sensor separation distance)	Bonferroni correction: 1.47 to 6.36
t	Linearity for static and dynamic across Code A and Code B, sensor distance 8 and 16 cm	Three-way ANOVA on linearity (factors: plume complexity code, and sensor separation distance)	Bonferroni correction: 0.044 to 0.086
u	Linearity for static and dynamic across Code A and Code B, sensor distance 8 and 16 cm	Three-way ANOVA on linearity (factors: plume complexity code, and sensor separation distance)	Bonferroni correction: 0.013 to 0.033
v	% success for static and dynamic across Code A and Code B, sensor distance 8 and 16 cm	Three-way ANOVA on % success (factors: plume complexity code, and sensor separation distance)	Bonferroni correction: 16.92 to 23.3
w	% success for static and dynamic across Code A and Code B, sensor distance 8 and 16 cm	Three-way ANOVA on % success (factors: plume complexity code, and sensor separation distance)	Bonferroni correction: 0.51 to 6.88
x	% success for static and dynamic across Code A and Code B, sensor distance 8 and 16 cm	Three-way ANOVA on % success (factors: plume complexity code, and sensor separation distance)	Bonferroni correction: 3.1 to 7.99

y	Linearity for static and dynamic across Code A and Code B, sensor distance 8 and 16 cm	Three-way ANOVA on linearity (factors: plume complexity code, and sensor separation distance)	Bonferroni correction: 0.13 to 0.17
z	Linearity for static and dynamic across Code A and Code B, sensor distance 8 and 16 cm	Three-way ANOVA on linearity (factors: plume complexity code, and sensor separation distance)	Bonferroni correction: 0.01 to 0.05
aa	Linearity for static and dynamic across Code A and Code B, sensor distance 8 and 16 cm	Three-way ANOVA on linearity (factors: plume complexity code, and sensor separation distance)	Bonferroni correction: 0.03 to 0.05
bb	% success for static and dynamic across Code A and Code B, sensor distance 8 and 16 cm	Three-way ANOVA on % success (factors: plume complexity code, and sensor separation distance)	Bonferroni correction: –16.23 to –9.86
cc	% success for static and dynamic across Code A and Code B, sensor distance 8 and 16 cm	Three-way ANOVA on % success (factors: plume complexity code, and sensor separation distance)	Bonferroni correction: –4.49 to 1.88
dd	% success for low complexity and high complexity across modalities (mouse, model Code A, model Code B, and robot Code B)	Two-way ANOVA on % success (factors: plume complexity and modality)	Bonferroni correction: –46.6 to –10.68
ee	% success for low complexity and high complexity across modalities (mouse, model Code A, model Code B, and robot Code B)	Two-way ANOVA on % success (factors: plume complexity and modality)	Bonferroni correction: –46.07 to –10.15
ff	% success for low complexity and high complexity across modalities (mouse, model Code A, model Code B, and robot Code B)	Two-way ANOVA on % success (factors: plume complexity and modality)	Bonferroni correction: –42.8 to –6.87
gg	% success for low complexity and high complexity across modalities (mouse, model Code A, model Code B, and robot Code B)	Two-way ANOVA on % success (factors: plume complexity and modality)	Bonferroni correction: –37.19 to –1.24
hh	Time to target for low complexity and high complexity across modalities (mouse, model Code A, model Code B, and robot Code B)	Two-way ANOVA on time to target (factors: plume complexity and modality)	Bonferroni correction: –44.17 to –23.34
ii	Time to target for low complexity and high complexity across modalities (mouse, model Code A, model Code B, and robot Code B)	Two-way ANOVA on time to target (factors: plume complexity and modality)	Bonferroni correction: –47.01 to –26.18
jj	Time to target for low complexity and high complexity across modalities (mouse, model Code A, model Code B, and robot Code B)	Two-way ANOVA on time to target (factors: plume complexity and modality)	Bonferroni correction: –45.67 to –24.84
kk	Time to target for low complexity and high complexity across modalities (mouse, model Code A, model Code B, and robot Code B)	Two-way ANOVA on time to target (factors: plume complexity and modality)	Bonferroni correction: –49.43 to –28.18
ll	Paired % success (no honeycomb condition vs honeycomb Code A), *n* = 4 sessions	Paired two-tailed *t* test	–97.78 to –27.22
mm	Paired % success (no honeycomb condition vs honeycomb Code B), *n* = 4 sessions	Paired two-tailed *t* test	–27.38 to –11.91
nn	Paired % success (no honeycomb condition vs honeycomb Code B), *n* = 4 sessions	Paired two-tailed *t* test	–67.52 to –27.48
oo	% success for honeycomb condition per start angle	One-way ANOVA (factor: start angle)	Bonferroni correction: 24.45 to 125.5
pp	% success for honeycomb condition per start angle	One-way ANOVA (factor: start angle)	Bonferroni correction: –6.11 to 116.1
qq	% success for honeycomb condition per start angle	One-way ANOVA (factor: start angle)	Bonferroni correction: 11.79 to 133.2
rr	% success for honeycomb condition per start angle	One-way ANOVA (factor: start angle)	Bonferroni correction: –19.37 to 114.4
ss	Linearity for honeycomb and no honeycomb using Code B across start angle	Two-way ANOVA (factors: plume complexity start angle)	Bonferroni correction: 0.051 to 0.29
tt	Linearity for honeycomb and no honeycomb using Code B across start angle	Two-way ANOVA (factors: plume complexity start angle)	Bonferroni correction: 0.047 to 0.32

uu	Linearity score for low complexity and high complexity across modalities (mouse, model Code A, model Code B, and robot Code B)	Two-way ANOVA on linearity score (factors: plume complexity and modality)	Bonferroni correction: 0.014 to 0.42
vv	Linearity score for low complexity and high complexity across modalities (mouse, model Code A, model Code B, and robot Code B)	Two-way ANOVA on linearity score (factors: plume complexity and modality)	Bonferroni correction: 0.046 to 0.45
ww	% success for low complexity and high complexity across modalities (mouse, model Code A, model Code B, and robot Code B)	Two-way ANOVA on % success (factors: plume complexity and modality)	Bonferroni correction: –36.2 to 14.06
xx	% success for low complexity and high complexity across modalities (mouse, model Code A, model Code B, and robot Code B)	Two-way ANOVA on % success (factors: plume complexity and modality)	Bonferroni correction: –48.87 to 1.39
yy	Time to target for low complexity and high complexity across modalities (mouse, model Code A, model Code B, and robot Code B)	Two-way ANOVA on time to target (factors: plume complexity and modality)	Bonferroni correction: –46.91 to –26.07
zz	Time to target for low complexity and high complexity across modalities (mouse, model Code A, model Code B, and robot Code B)	Two-way ANOVA on time to target (factors: plume complexity and modality)	Bonferroni correction: –51.97 to –31.13
aaa	Velocity for low complexity and high complexity across modalities (mouse, model Code A, model Code B, and robot Code B)	Two-way ANOVA on time to target (factors: plume complexity and modality)	Bonferroni correction: 16.77 to 25.09
bbb	Velocity for low complexity and high complexity across modalities (mouse, model Code A, model Code B, and robot Code B)	Two-way ANOVA on time to target (factors: plume complexity and modality)	Bonferroni correction: 24.9 to 33.22

## Results

### Mice successfully locate odor source within a non-turbulent chaotic flow chamber

To test mouse navigation within an airborne odor plume, we built a 1 × 1 × 0.3 m flow chamber behavioral arena based on that used by Connor et al. (2018). We introduced two honeycombs on either end to laminarize the airflow established by a vacuum at the outlet end. To generate a controlled complex odor plume within this flow chamber, we inserted a turbulence grid in front of the honeycomb at the inlet end (Fig. 1*A*). A flow rate of 5 cm/s was established within the flow chamber. For the purposes of this study, we refer to this flow chamber as a SOL. Three odor ports at the inlet end of the flow chamber released odor, generating plumes. We measured the time averaged concentration of odor across the flow chamber within each of the three plumes using a miniature photoionization detector, miniPID (Fig. 1*C*).

We trained a group of mice on a task to navigate to the source of these airborne odor plumes within the SOL. On any given trial, an odor plume was established from one of the three odor ports for 30 s before the insertion of the animal into the behavioral arena. The task structure required water-regulated mice to locate an odor port releasing IAA (3% in mineral oil) within 45 s to receive a sucrose water reward from an adjacent lick spout (Fig. 1*B*; Movie 3). Other studies aimed at understanding rodent navigation within airborne odor plumes have found that with experience animals preferentially use a localization strategy in which they serially explore all possible odor source locations, showing a shift away from using solely odor-based cues (Bhattacharyya and Bhalla, 2015; Gire et al., 2016). To ensure that the mice in this study relied only on odor information, we terminated trials when the mouse reached one of three odor ports, providing water reward only if the odor-releasing port (i.e., not the two clean air-releasing ports) was reached. This behavioral design incentivizes mice to make a decision regarding odor source location, rather than testing all possible sources.

Movie 3. Mouse navigation to airborne odor source. In first trial animal, odor port 3 is releasing odor. In second trial, odor port 2 is releasing odor. Video recorded and played back at 15 Hz. 10.1523/ENEURO.0212-19.2019.video.3

Before being tested on this task, animals were trained to associate the localization of an odor port releasing odor with delivery of a sucrose water reward. Animals were able to learn the task following a 6 d of this training and performed consistently above chance starting the eight day of testing (Fig. 1*D*; one-tailed two-sample *t* test with Holm–Sidak correction for multiple comparisons, *p* = 0.047 for day 6, *p* = 0.047 for day 8, *p* = 0.0026 for day 9, *p* = 0.0013 for day 10, *p* = 0.018 for day 11, *p* = 0.033 for day 12, *p* = 0.0026 for day 13, *p* = 0.047 for day 14, *n* = 4 mice). Thus, the testing days were classified into two phases of 7 d each, the early phase and the late phase. Thigmotaxis (wall-hugging) behavior indicates an anxiety-like state in mice. Mice decreased the percentage of the 45-s trial spent engaging in wall-hugging behavior over time (Fig. 1*E*; paired one-tailed *t* test, late phase vs early phase difference: –27.03 ± 2.79, *p* = 0.0012, *n* = 4 mice^a^).

### Mouse performance remains robust with increased complexity but shows a shift in strategy

To test the effect of increased complexity on odor localization performance, we removed the honeycomb at the inlet side of the flow chamber (Extended Data Fig. 1-1). This allows for the introduction of ambient air complexity into the behavioral arena in addition to that caused by the turbulence grid. We refer to this odor environment as “non-turbulent chaotic” as well as “complex.” When comparing the two environments, we refer to the honeycomb condition interchangeably with “low-complexity” and the no honeycomb condition with “high-complexity” environments. The SD s of the 2-min odor concentration time series at each midline downstream location (six repeats each) were all significantly increased by roughly two- to four-fold (3.9, 2.3, 1.8, and 2.1 times the SD with inlet honeycomb at 10, 30, 50, and 60 cm downstream from the odor tube, respectively). The SD normalized by mean odor concentration was also significantly increased at 10 and 30 cm from the odor tube by 4.0- and 1.9-fold, respectively. Note that instrument noise contribution to the SD was negligible.

**Figure 2.**
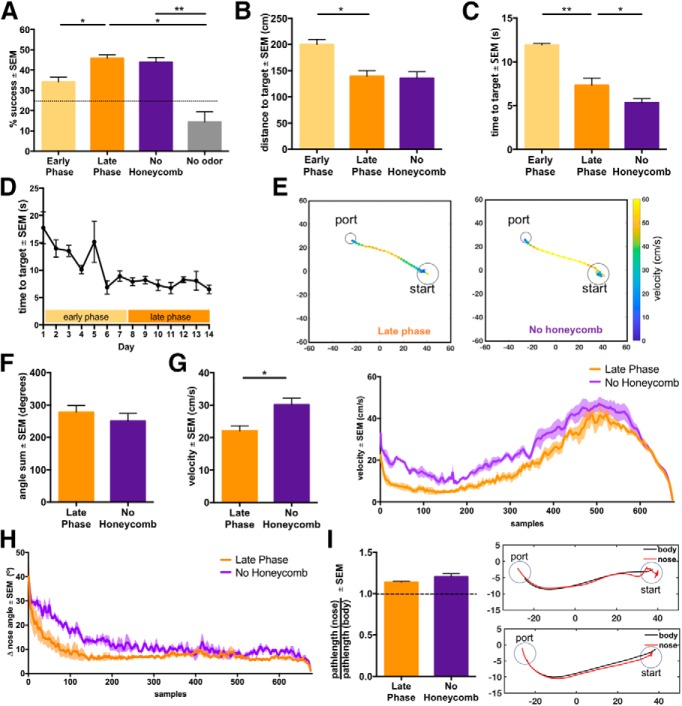
Mice change navigation behavior with increased experience and odor environment complexity. ***A***, Performance (average % successful trials over sessions) across testing phases. Mice are tested on a no-odor condition in addition to the phases with a honeycomb and condition without a honeycomb. Chance level performance is 25% as animals have three ports as options and are not required to choose an odor port on trials. ***B***, Pathlength to target odor port on successful trials. ***C***, Time to target odor port on successful trials. ***D***, Time to target on successful trials over testing days. ***E***, Example traces of successful navigation from the late phase and no honeycomb phase. Traces are color scaled based on velocity. ***F***, Total angle sum of trajectories of late phase and no honeycomb condition. Total angle sum is calculated by using the total sum of angles on turns from frame-to-frame. ***G***, Velocity on successful trials of late phase and honeycomb condition (left). Velocity over the course of successful trajectories resampled to 675 frames (right). ***H***, Change in nose angle per frame (15 Hz) over the course of successful trajectories resampled to 675 frames (left). Change in nose angle on successful trials of late phase and no honeycomb condition (right). ***I***, Ratio of path distance based on nose to path distance based on center of body (left). Example trajectories with ratios of 1.35 (top) and 1.08 (bottom). All plots show mean ± SEM, *n* = 4 mice. See also Extended Data Figure 2-1. *p < 0.05, **p < 0.01.

Animals perform at a significantly higher % success in the late phase when compared to the early phase and show no change in performance between the late phase and no honeycomb condition (Fig. 2*A*; paired *t* test one-tailed, late phase vs early phase difference = 11.65 ± 3.1%, *p =* 0.016^b^, paired *t* test two-tailed, no honeycomb vs late phase difference = –1.92 ± 2.74%, *p* = 0.53^c^, *n* = 4 mice). This shows a significant improvement of performance over time in the same odor environment and that with increased odor plume complexity animals show consistent task performance. Additionally, no difference in performance is seen across ports between the late phase and the no honeycomb condition, although there was a small effect of port number (Extended Data Fig. 2-1*A*; two-way ANOVA, main effect of plume complexity, *p* = 0.8, main effect of port = 0.039, *n* = 4 mice). This effect of port number may be because the animals were lick-trained on odor port 1 (although *post hoc t* tests with Bonferroni correction for multiple comparisons do not reveal a significant difference between ports, port 1 vs port 2 difference: 26.2 ± 10.23%, *p* = 0.0917^d^, port 1 vs port 3 difference: 28.35 ± 10.23%, *p* = 0.065^e^, port 2 vs port 3 difference: 37.67 ± 10.23%, *p* > 0.99^f^, *n* = 4 mice). To ensure that animals were using odor information for this task, we tested them on a set of ∼30 trials without odor between the late phase and no honeycomb condition. Animals performed at chance levels without odor and their performance was significantly lower than that during the late phase or no honeycomb phase (Fig. 2*A*; paired *t* test one-tailed, no odor vs late phase difference: –31.32 ± 6.24, *p* = 0.0076^g^, no odor vs no honeycomb difference: –29.4 ± 5.22, *p =* 0.0055^h^, *n* = 4 mice).

10.1523/ENEURO.0212-19.2019.f2-1 Extended Data Figure 2-1 Mice show consistent performance and turning behavior across both low-complexity and high-complexity odor environments. ***A***, % success of mouse navigation at each target odor port in the late phase and no honeycomb conditions. ***B***, Same as ***A***, for total angle sum. ***C***, Same as ***A***, for linearity score. All plots show mean ± SEM, *n* = 4 mice. Download Figure 2-1, TIF file.

We recorded behavior during trials using a camera placed above the flow chamber and imaged through the transparent lid of the behavioral arena. We found that on successful trials, the distance and time to the target odor port decreases between the early and late phase (Fig. 2*B–D*; paired *t* test two-tailed, distance to target of late phase vs early phase difference: –60.79 ± 16.8 cm, *p* = 0.036^i^, time to target of early phase vs late phase difference: –4.6 ± 0.73 s, *p* = 0.008^j^, *n* = 4 mice), showing that animals are taking shorter and faster routes to the correct odor port over time. Additionally, the early phase shows a significant negative linear trend of time to correct odor port over time, whereas the late phase does not show a significant decline. Thus, their behavior has stabilized when entering into the late phase (Fig. 2*D*; linear regression, *R* ^2^ = 0.62 early phase, *p* = 0.0357, *R* ^2^ = 0.006 late phase, *p* = 0.71, *n* = 4 mice).

We measured several parameters associated with the animals’ behavior during the trial, as the level of odor plume complexity could affect the path taken and parameters modulated during the animals’ trajectories. We found that when the honeycomb was removed and complexity was increased, the distance to the target on successful trials remained the same as the late phase, but the time to the target significantly decreased (Fig. 2*B*,*C*; paired *t* test two-tailed, distance to target no honeycomb vs late phase difference: –3.52 ± 7.05 cm, *p* = 0.65^k^, time to target no honeycomb vs late phase difference: –1.99 ± 0.57 s, *p* = 0.039^l^, *n* = 4 mice). Additionally, the animals traveled at a higher velocity when navigating a more chaotic plume (Fig. 2*E*,*G*; paired *t* test two-tailed, no honeycomb vs late phase difference: 8.044 ± 2.37 cm/s, *p* = 0.043^m^, *n* = 4 mice).

Casting involving lateral full-body or head movement during odor-based navigation is a behavioral strategy that has been extensively characterized and found to be conserved across several species (Vickers, 2000; Grasso, 2001). Invertebrates including moths, flies, and cockroaches implement this zig-zagging behavior when localizing odor within an airborne odor plume, particularly when attempting to reacquire the odor stream (David et al., 1983; Kennedy, 1983; Baker and Haynes, 1987; Kuenen and Cardé, 1994; Grasso, 2001; Cardé and Willis, 2008; Gomez-Marin et al., 2011; van Breugel and Dickinson, 2014). Additionally, mammals, including both rodents and humans, display lateral head movements when tracking odor trails (Porter et al., 2007; Khan et al., 2012; Catania, 2013). Here we measured “casting” using two parameters. The first is the path curvature as measured by the absolute total sum of turning angles during a trial. Animals did not display any difference in turning behavior between the late phase and no honeycomb condition (Fig. 2*F*; Extended Data Fig. 2-1*B*; paired *t* test two-tailed, no honeycomb vs late phase difference: –27.19 ± 13.39°, *p* = 0.14^n^, two-way ANOVA, total angle sum main effect of plume complexity, *p* = 0.92, total angle sum main effect of port number, *p* = 0.63; *n* = 4 mice). Average total sum of turning angles for both conditions are below 360°, and thus mouse turning behavior remains below a full rotation during navigation, suggesting minimal full-body casting. This lack of casting behavior is in alignment with previous observations in rodents navigating in odor plumes (Bhattacharyya and Bhalla, 2015; Gire et al., 2016). The second form of casting measured was the change in nose angle, thereby measuring sweeps in head movement during odor localization. We found that mice show modest changes in nose angle which are slightly higher when the chaotic nature of the odor plume is increased (Fig. 2*H*; paired *t* test two-tailed, no honeycomb vs late phase difference: 2.94 ± 0.83°, *p* = 0.04^o^, *n* = 4 mice). Additionally, the ratio of the trial pathlength as measured by the nose position to that measured by the body position shows that nose pathlength is greater than body pathlength (Fig. 2*I*; one-sample *t* test, μ = 1, late phase mean: 1.14 ± 0.004, *p* < 0.0001^p^, no honeycomb mean: 1.20 ± 0.02, *p =* 0.0016^q^, *n* = 4 mice). Thus, this suggests that mice do not display lateral body movements, but do exhibit sweeping movements with their head during odor plume navigation. However, these head movements appear to be limited to the initial phase of olfactory search behavior (Fig. 2*H*).

Interestingly, trajectories from one test session show few differences between the late phase and no honeycomb condition (Fig. 5*A*). Additionally, animals’ path linearity, as measured by the fraction of distance of a straight-line path to that of the actual path, did not vary across rewarded ports, showing consistency across tested plumes (Extended Data Fig. 2-1*C*; two-way ANOVA, linearity main effect of plume complexity, *p* = 0.81, linearity main effect of port number, *p* = 0.9, *n* = 4 mice). Overall, these results suggest that increased odor plume complexity does not affect odor navigation performance. However, animals do alter their strategy when navigating a more chaotic plume, where a faster speed may be beneficial for odor localization, whereas modulating parameters that affect trajectory structure may not be as important.

### Model-based odor navigation

**Figure 3.**
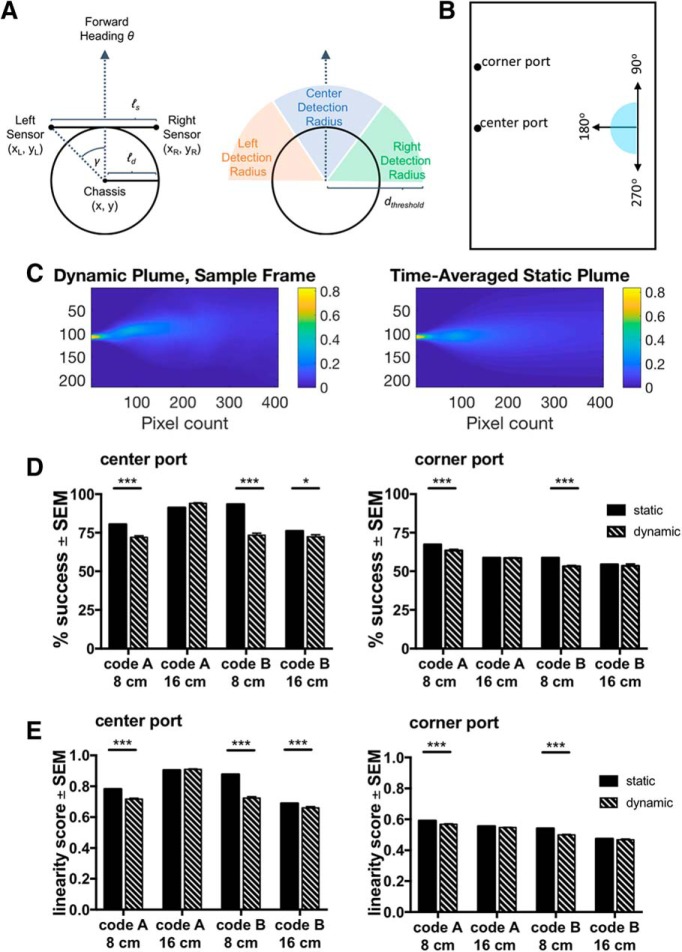
*In silico* models show decreased performance with increased odor environment complexity. ***A***, Model virtual chassis moves through space with a heading, *θ*. Two sensors are separated at a distance *ℓ_s_*and an angle γ (left). If the center of the model reaches d_threshold_ = 10 cm of the wall, the model will take corrective measures (right; described in Materials and Methods). ***B***, Model is tested at angles ranging from 90° to 270° with a start position in the center of the arena. Model is tested on two plumes, one originating from a center port and one from a corner port. ***C***, Sample frame depicting instantaneous concentration of the dynamic plume normalized to odor source (left), and an image of the stationary concentration gradient in static plume normalized to odor source (right). ***D***, Performance (average % success of all start angles ± SEM) across code and sensor distance for center target port (left) and corner target port (right); *n* = 20 simulations. ***E***, Linearity score (calculated as the ratio of the Euclidean distance between start point and end point of trajectory and the actual pathlength) across code and sensor distance for center target port (left) and corner target port (right). Plot shows mean linearity score ± SEM, *n* = 20 simulations. See also Extended Data Figures 3-1, 3-2, 3-3, 3-4, 3-5, and 3-6. *p < 0.05, **p < 0.01, ***p < 0.001.

To compare mouse odor navigation with simple odor localization algorithms, we developed an *in silico*-simulated robot. The simulated robot has two odor sensors, with a separation distance that can be varied, and can make comparisons between the odor signals at the left and right sensor. It has a virtual frame and moves through a virtual odor plume with a heading *θ*. If the simulated robot approaches the wall of the virtual arena, it will take corrective measures to reorient toward the open arena (Fig. 3*A*). We tested this *in silico* model in a virtual SOL with a center and corner port, analogous to that in which we tested the mice (Fig. 3*B*). We tested the simulated robot navigation starting at the center of the arena with start angles varying from 90^°^ to 270^°^ at 3.6^°^ increments. Acetone PLIF data were used as the odor plume input for the virtual arena, obtained from Connor et al. (2018). To assess the effect of odor plume complexity on the behavior of our model, we tested the simulated robot using either a static odor plume (i.e., the average of 4 min of odor plume data) or using a dynamic odor plume with real-time fluctuations (Fig. 3*C*).

10.1523/ENEURO.0212-19.2019.f3-1 Extended Data Figure 3-1 Instantaneous concentration for *in silico* algorithm Code A at center start position over trajectories resampled to 755 frames. Each trajectory was resampled to 755 frames (the maximum amount of time the model was allotted) and averaged across starting angle (*y*-axis). Twenty simulations per starting angle were tested. Concentration shown with color scale. For first ∼275 samples, the model is stationary due to collecting baseline data, thus the odor concentration does shows little variation during this sampling period. Data are grouped by left and right sensor reading, tested odor plume (static or dynamic), and sensor separation distance (8 and 16 cm). For each condition, the average concentration at each starting angle is plotted as well as the SD of concentration on these trajectories. Download Figure 3-1, TIF file.

10.1523/ENEURO.0212-19.2019.f3-2 Extended Data Figure 3-2 Instantaneous concentration for *in silico* algorithm Code A at corner start position over trajectories resampled to 755 frames. Each trajectory was resampled to 755 frames (the maximum amount of time the model was allotted) and averaged across starting angle (*y*-axis). Twenty simulations per starting angle were tested. Concentration shown with color scale. For first ∼275 samples, the model is stationary due to collecting baseline data, thus the odor concentration does shows little variation during this sampling period. Data are grouped by left and right sensor reading, tested odor plume (static or dynamic), and sensor separation distance (8 and 16 cm). For each condition, the average concentration at each starting angle is plotted as well as the SD of concentration on these trajectories. Download Figure 3-2, TIF file.

10.1523/ENEURO.0212-19.2019.f3-3 Extended Data Figure 3-3 Instantaneous concentration for *in silico* algorithm Code B at center start position over trajectories resampled to 755 frames. Each trajectory was resampled to 755 frames (the maximum amount of time the model was allotted) and averaged across starting angle (*y*-axis). Twenty simulations per starting angle were tested. Concentration shown with color scale. For first ∼275 samples, the model is stationary due to collecting baseline data, thus the odor concentration does shows little variation during this sampling period. Data are grouped by left and right sensor reading, tested odor plume (static or dynamic), and sensor separation distance (8 and 16 cm). For each condition, the average concentration at each starting angle is plotted as well as the SD of concentration on these trajectories. Download Figure 3-3, TIF file.

**Figure 4.**
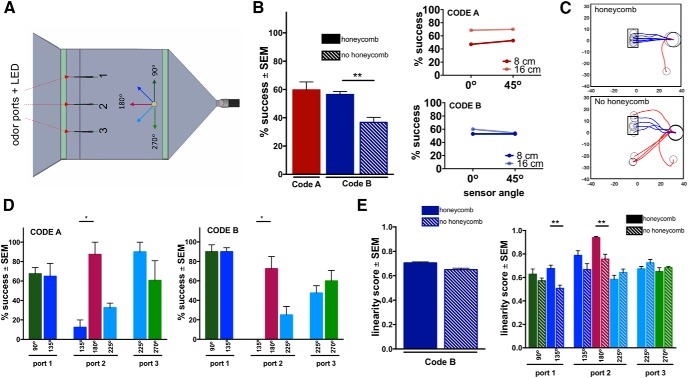
Arduino-based robot navigation varies based on start position and odor environment complexity. ***A***, Robot odor navigation flow chamber, modifications to the SOL. Solid arrows represent five starting angles. Odor ports were coupled to LED lights detected by sensors on the robot (indicated by dotted red arrows). ***B***, Performance (average % successful trials over 8 and 16 cm and 0° and 45° gas sensor distance and angles, respectively) across codes (left). Performance based on gas sensor distance and angle for the honeycomb condition (right). ***C***, Example trajectories from 180° (magenta) starting position in ***A*** for honeycomb and no honeycomb condition. ***D***, Performance (average % successful trials over 8 and 16 cm and 0° and 45° gas sensor distance and angles, respectively) with the honeycomb based on starting angle and rewarded port for Code A (left) and Code B (right). Bars are color coded and labeled according to the starting angles in ***A***. ***E***, Robot overall linearity score with honeycomb and without honeycomb using Code B. Plot shows data combined over sensor angle and sensor distance for each odor environment condition (left). Linearity score across starting angles and target ports with and without the honeycomb. All plots show mean ± SEM, *n* = 4 sessions. See also Extended Data Figure 4-1. *p < 0.05, **p < 0.01.

10.1523/ENEURO.0212-19.2019.f3-4 Extended Data Figure 3-4 Instantaneous concentration for *in silico* algorithm Code B at corner start position over trajectories resampled to 755 frames. Each trajectory was resampled to 755 frames (the maximum amount of time the model was allotted) and averaged across starting angle (*y*-axis). Twenty simulations per starting angle were tested. Concentration shown with color scale. For first ∼275 samples, the model is stationary due to collecting baseline data, thus the odor concentration does shows little variation during this sampling period. Data are grouped by left and right sensor reading, tested odor plume (static or dynamic), and sensor separation distance (8 and 16 cm). For each condition, the average concentration at each starting angle is plotted as well as the SD of concentration on these trajectories. Download Figure 3-4, TIF file.

**Figure 5.**
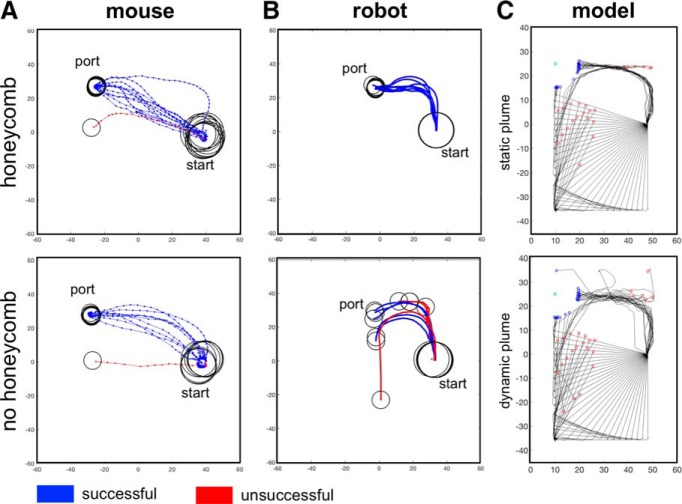
Mouse, robot, and *in silico* navigation trajectories. ***A***, Mouse trajectories show consistency with increased odor environment complexity. ***B***, Robot trajectories show decreased success on trials for the same testing conditions with increased odor plume complexity, Code B, sensor distance: 8 cm, sensor angle: 0°. ***C***, *In silico* trajectories (50 trials with start angles ranging from 90° to 270°) show increased unsuccessful trials for the same testing conditions with increased complexity, Code B, sensor distance: 8 cm. See also Extended Data Figure 5-1.

10.1523/ENEURO.0212-19.2019.f3-5 Extended Data Figure 3-5 Schematics of model trajectories through odor plume. ***A***, Example model trajectory through center odor plume. Striated patterning seen in Extended Data Figures 3-1, Figures 3-4 is due to robot rotating, causing sensors to rotate in and out of the odor plume. Striated patterning is more obvious at 16-cm sensor separation distance due to sensors being wider apart and therefore detecting odor environments with greater concentration differences. Additionally, striated patterning is less obvious in the dynamic plume because the plume is dynamic and the paths are not deterministic, so averages across trials will show a smoother gradient of concentration over trial time. ***B***, Example model trajectory through corner odor plume. Model begins out of the odor plume, and therefore, the first several frames in Extended Data Figure 3-3, Figure 3-4 show a very low concentration. Again, striated patterning is more obvious at 16-cm sensor separation distance and less obvious in the dynamic plume condition. Download Figure 3-5, TIF file.

10.1523/ENEURO.0212-19.2019.f3-6 Extended Data Figure 3-6 Navigation performance and trajectory linearity across start angles. ***A***, % success (mean performance of one simulation with all start angles tested) and linearity score with static and dynamic plume using binaral model (Code A) and temporal-based binaral model (Code B) across starting angles with a sensor separation distance of 8 cm. Graphs are grouped target port location (either center port or corner port). Plots show mean % success ± SEM or mean linearity score ± SEM; *n* = 20 simulations, Code A shown in red, Code B shown in blue. ***B***, Same as ***A***, for a sensor separation distance of 16 cm. Download Figure 3-6, TIF file.

We created two navigational algorithms to test *in silico* odor localization. These algorithms were designed to incorporate a minimal interpretation of stereo smell while, in one case, also incorporating features to resolve the fluctuating nature of our odor plume. For both algorithms a baseline reading is collected for each sensor as the average of four readings over 1 s. These two baselines are then averaged to be used for odor-based navigation. In the first algorithm, which we refer to as Code A, if the difference between the instantaneous sensor reading at the left sensor and the right sensor, both corrected for the baseline reading, is greater than the threshold (described in Materials and Methods), the model turns left and moves forward for a subsequent reading. If the difference between the right sensor and the left sensor reading, corrected for the baseline, is greater than the threshold, the model turns right and advances. If neither of these conditions are true, the model moves forward.

The most basic model implemented in a robotics approach aimed at odor plume tracking is one in which the robot with a pair of chemical sensors simply moves in the direction of higher concentration. However, this approach may be limited due to the previously described dynamic nature of odor plumes in which the robot can at one moment sense odor that quickly disappears while remaining stationary (Sandini et al., 1993; Kazadi et al., 2000; Lilienthal and Duckett, 2004; Ishida et al., 2012). Models that rely on averaging several frames on odor intake before determining movement may be more successful at determining concentration gradients (Ishida et al., 2001). Using this logic, we created Code B. In this algorithm, if the difference between the average (of the readings of the two sensors) across two time points is greater than a threshold, the model will move forward, as this indicates the simulated robot is moving up the concentration gradient. Otherwise, Code B defaults to the same rules described for Code A.

### *In silico*-simulated robot navigation is affected by increased plume complexity

As previously mentioned, stereo smell is important for odor navigation in both mammals and invertebrates. The distance between olfactory sensors may play a role in the ability of an animal to accurately detect an odor plume and locate the source. We tested the simulated robot in both the static and dynamic odor plumes with two sensor separation distances, 16 and 8 cm. Model Code A performs at a significantly lower success rate in the presence of increased plume complexity at an 8-cm sensor separation distance regardless of active odor port position (Fig. 3*D*; Extended Data Fig. 3-6; two-tailed *t* test center port Code A 8-cm static vs center port Code A 8-cm dynamic difference: 8.37 ± 1.1%, *p* < 0.0001^r^, two-tailed *t* test corner port Code A 8-cm static vs corner port Code A 8-cm dynamic difference: 3.91 ± 0.84%, *p* < 0.0001^s^, *n* = 20 simulations). Additionally, Code A at 8 cm shows a decrease in trajectory linearity as an average and across starting angles when the plume complexity increases, suggesting that with increased complexity, paths become more winding (Fig. 3*E*; Extended Data Fig. 3-6; two-tailed *t* test center port Code A 8-cm static vs center port Code A 8-cm dynamic difference: 0.065 ± 0.007, *p* < 0.0001^t^, two-tailed *t* test corner port Code A 8-cm static vs corner port Code A 8-cm dynamic difference: 0.023 ± 0.0035, *p* < 0.0001^u^, *n* = 20 simulations). Model Code B shows a significant decrease in performance with increased plume complexity at a 16-cm sensor separation distance with a center odor plume and an 8-cm sensor separation distance regardless of plume position (Fig. 3*D*; Extended Data Fig. 3-6; two-tailed *t* test center port Code B 8-cm static vs center port Code B 8-cm dynamic difference: 20.11 ± 1.1%, *p* < 0.0001^v^, center port Code B 16-cm static vs center port Code B 16-cm dynamic difference: 3.70 ± 1.1%, *p* = 0.011^w^, corner port Code B 8-cm static vs corner port Code B 8-cm dynamic difference: 5.54 ± 0.84%, *p* < 0.0001^x^, *n* = 20 simulations). Data from both codes show that at an 8-cm sensor separation distance, algorithms are more susceptible to a decrease in performance due to increased odor plume complexity.

Additionally, linearity as an average and across starting angles for Code B decreases with increasing plume complexity, indicating that with either sensor separation distance, paths become less linear with increased complexity (Fig. 3*E*; Extended Data Fig. 3-6; two-tailed *t* test center port Code B 8-cm honeycomb vs center port Code B 8-cm no honeycomb difference: 0.15 ± 0.007, *p* < 0.0001^y^, center port Code B 16-cm honeycomb vs center port Code B 16-cm no honeycomb difference: 0.03 ± 0.007, *p* = 0.0006^z^, corner port Code B 8-cm honeycomb vs corner port Code B 8-cm no honeycomb difference: 0.042 ± 0.003, *p* < 0.0001^aa^, *n* = 20 simulations). Trajectories within the static odor plume are deterministic as there is a fixed odor plume gradient to climb, whereas there was variation in the paths within the dynamic plume, as expected (Extended Data Figs. 3-1, 3-2, 3-3, 3-4, 3-6; Movies 4, 5, 6, 7, 8, 9, 10, and 11). Interestingly, both the success and linearity of Code B at an 8-cm separation distance in the dynamic plume shows periodicity where the success and linearity decrease and rise every 30° of starting angles (Extended Data Fig. 3-6). This periodicity may be attributed to the 30° turn angle implemented *in silico* and if the simulated robot is capable of rotating to 180° (facing the odor source) using the increment, it will ultimately be more successful and have a straighter path.

Movie 4. In silico model navigation of static odor plume released from corner odor port using Code A. Video recorded at 10 Hz and played back at 60 Hz. 10.1523/ENEURO.0212-19.2019.video.4

Movie 5. In silico model navigation of static odor plume released from center odor port using Code A. Video recorded at 10 Hz and played back at 60 Hz. 10.1523/ENEURO.0212-19.2019.video.5

Movie 6. In silico model navigation of dynamic odor plume released from corner odor port using Code A. Video recorded at 10 Hz and played back at 60 Hz. 10.1523/ENEURO.0212-19.2019.video.6

Movie 7. In silico model navigation of dynamic odor plume released from center odor port using Code A. Video recorded at 10 Hz and played back at 60 Hz. 10.1523/ENEURO.0212-19.2019.video.7

Movie 8. In silico model navigation of static odor plume released from corner odor port using code B. Video recorded at 10 Hz and played back at 60 Hz. 10.1523/ENEURO.0212-19.2019.video.8

Movie 9. In silico model navigation of static odor plume released from center odor port using code B. Video recorded at 10 Hz and played back at 60 Hz. 10.1523/ENEURO.0212-19.2019.video.9

Movie 10. In silico model navigation of dynamic odor plume released from corner odor port using code B. Video recorded at 10 Hz and played back at 60 Hz. 10.1523/ENEURO.0212-19.2019.video.10

Movie 11. In silico model navigation of dynamic odor plume released from center odor port using code B. Video recorded at 10 Hz and played back at 60 Hz. 10.1523/ENEURO.0212-19.2019.video.11

When comparing performance across codes, in the static condition, Code A had a significantly lower % success than Code B at an 8-cm sensor separation distance, however Code B performed significantly worse than Code A at a 16-cm sensor separation distance, showing the interaction between code and sensor separation distance (Fig. 3*D*; two-tailed *t* test center port Code A 8-cm static vs Code B 8-cm static difference: –13.04 ± 1.1%, *p* < 0.0001, center port Code A 16-cm static vs Code B 16-cm static difference: 15.22 ± 1.1%, *p* < 0.0001^bb^, *n* = 20 simulations). In the dynamic condition, just as in the static condition, Code A performs significantly better than Code B at a 16-cm sensor separation distance (Fig. 3*D*; Code A 16-cm turbulent vs Code B 16-cm turbulent difference: 21.63 ± 1.1%, *p* < 0.0001^cc^, *n* = 20 simulations). Together, these findings suggest that with a small sensor separation distance Code B is more successful, however at a larger sensor separation distance Code A is more successful.

Difference in trajectories between static and dynamic conditions can be observed in Figure 5*C*. Our simulated robot was tested using data collected in the SOL at the same starting position as the mice, therefore we can directly compare performance between the two. Model Code A overall performs with a higher % success than the mice, but there is no significant difference between performance of model code B and the mice [Extended Data Fig. 5-1*A*, left; two-tailed *t* test low-complexity mouse vs Code A difference: –25.68 ± 8.74%, *p* = 0.043^dd^, high-complexity mouse vs Code A difference: –25.38 ± 8.74%, *p* = 0.048^ee^, low-complexity mouse vs Code B difference: –21.68 ± 8.74%, *p* = 0.12^ff^, high-complexity mouse vs Code B difference: –16.63 ± 8.74%, *p* = 0.42^gg^, *n* = 4 mice, *n* = 4 sessions for each model condition (one session for per combination of sensor distance and target odor port)]. Additionally, mice locate the odor source on successful trials significantly faster than both codes [Extended Data Fig. 5-1*B*, two-tailed *t* test low-complexity mouse vs Code A difference: –33.75 ± 3.63 s, *p* < 0.0001^hh^, high-complexity mouse vs Code A difference: –36.59 ± 3.63 s, *p* < 0.0001^ii^, low-complexity mouse vs Code B difference: –35.25 ± 3.63, *p* < 0.0001^jj^, high-complexity mouse vs Code B difference: –39.01 ± 3.63 s, *p* < 0.0001^kk^, *n* = 4 mice, *n* = 4 sessions for each model condition (one session for per combination of sensor distance and target odor port)]. These findings show that although the Code A outperforms a mouse in terms of % success for the low and high plume complexity conditions, both codes show a decrease in within code performance in the presence of increased complexity, a behavioral shift not seen in mice.

### Arduino-based robot shows decrease in performance with increased odor plume complexity

To test how our *in silico* models perform in a real flow chamber, we tested an Arduino-based robot using Code A and Code B in the previously described SOL behavioral arena. We modified the arena to replace lick spouts with LEDs associated with each odor port which were detected by light sensors on the robot to identify if an odor port had been approached. The Arduino-based robot was equipped with optimized gas sensors attached to a fan that actively sucked air through the sensors. In addition, we attached proximity sensors to avoid contact with the walls of the flow chamber. The gas sensors were optimized for response speed by removing the front of steel mesh cap surrounding the front of the sensor, drilling a hole through the pc-board behind the sensor and fitting a small fan on the back of the hole (Extended Data Fig. 4-1*A*). The responsiveness of the sensor was improved by an order of magnitude: time from stimulus onset (i.e., the first time the signal crosses 2% of peak amplitude) to 75% of peak (t75 O) was 0.67 s in the unmodified sensor but reduced to 0.07 s when modified, being 1.13 s and 0.11 s (t100 O) to reach peak value, respectively (Extended Data Fig. 4-1*C*). Decay time from peak to 50% of peak (t50 off) was reduced from 2.41 to 0.47 s, and to 25% of peak (t25 off) from 4.96 to 2.14 s, respectively. The distance between these gas sensors could be varied, as well as the angle at which they were oriented.

10.1523/ENEURO.0212-19.2019.f4-1 Extended Data Figure 4-1 Increased odor plume complexity impairs Arduino-based robot navigation from alternate starting position. ***A***, Top and side view of robot with three proximity, two VOC gas sensors with fans, and an LED sensor. ***B***, ***C***, Normalized odor concentration reading after brief ethanol exposure over time with an original sensor powered at 5 V (1.25 W per sensor), a modified sensor with fan at 6.5 V (2 W) without driving the fan, and a modified sensor with fan at 6.5 V and driving the fan using 3 V (0.15 W). t50 on P: rise time from t50 (time at 50% of peak amplitude) to tp (peak amplitude). t50 off: decay time from to tp to t50. t25 on P: rise time from t25 (25% of peak amplitude) to tp (peak amplitude). t25 off: decay time from to tp to t25. t75 on O: rise time from response onset (2% of peak amplitude) to t75 (75% of peak amplitude). t100 on O: rise time from response onset (2% of peak amplitude) to t100 (peak amplitude). ***D***, Robot odor navigation flow chamber. Red arrow labeled “start” indicates the alternate starting position and the red asterisk indicates the active odor port. ***E***, Performance (average % successful trials over 8 and 16 cm and 0° and 45° gas sensor distance and angles, respectively) across codes with and without honeycomb. Plot shows mean % success ± SEM, *n* = 4 sessions (left). Performance based on gas sensor distance (8 and 16 cm) and angle (0° and 45°) for the honeycomb and no honeycomb conditions (right). Download Figure 4-1, TIF file.

We tested the robot starting on the midline of the outlet end of the flow chamber for direct comparison with mouse and *in silico* model behavior. We used six different starting angles with varying active odor ports based on starting angle (Materials and Methods; Fig. 4*A*). At this starting position, we tested the robot using Code A and Code B with the honeycomb as well as Code B without the honeycomb. Additionally, we recorded behavior at an alternate start position, which cannot be directly compared to the mouse behavior, in which the start angle of the robot was 270° at the far-right corner of the outlet end of the chamber. In this condition the center port was used for plume generation (Extended Data Fig. 4-1*D*). At this start position, we tested the robot using both Code A and Code B with and without the honeycomb. At both starting positions we tested the robot with sensor separation distances of 8 and 16 cm and sensor angles of 0°, parallel with the front of the robot, and 45°. Additionally, we tested the robot using 70% ethanol instead of IAA, used with mice, to obtain robust odor readings from the robot’s gas sensors. The task structure for the robot odor-based navigation was nearly identical to that of the mouse; however, the robot was allotted 75 s to reach the odor source.

We studied how the behavior of the robot changed when tested with the two algorithms in the presence of increased complexity by removing the honeycomb at the inlet side of the flow chamber, the exact conditions we tested on the mice. Code A showed a decrease in performance at the corner start position when the honeycomb was removed and Code B show a significant decrease in % success with increased complexity at both start positions (Fig. 4*B*, left, *C*; Extended Data Fig. 4-1*E*, left; paired two-tailed *t* test, corner start Code A no honeycomb vs Code A with honeycomb difference: –62.5 ± 11.09%, *p* = 0.011^ll^, center start Code B no honeycomb vs Code B with honeycomb difference: –19.64 ± 2.43%, *p* = 0.004^mm^, corner start Code B no honeycomb vs Code B with honeycomb difference: –47.5 ± 6.29%, *p* = 0.0048^nn^, *n* = 4 sessions). Additionally, when implementing Code A with the honeycomb, the robot shows a higher success rate at a greater sensor separation for both sensor angles at a center start position and at a 0^°^ sensor angle at a corner start position (Fig. 4*B*, right; Extended Data Fig. 4-1*E*, right). A larger sensor separation distance may be beneficial for the robot navigation using Code A because larger spatial differences in the concentration gradient can be detected. This finding is in line with that of the *in silico* model.

Performance of the robot also varies based on starting angle. When the center port is active, the robot performs at a higher % success when oriented directly toward the source than when angled 45° away from the source (Fig. 4*D*, one-way ANOVA port 2, Code A effect of start angle, *p* = 0.0021, two-tailed *t* test, Code A 180° vs Code A 135° difference: 75 ± 10.41%, *p* = 0.017^oo^, Code A 180° vs Code A 225° difference: 55 ± 12.58%, *p* = 0.067^pp^, Code B effect of start angle, *p* = 0.0055, Code B 180° vs Code B 135^°^ difference: 72.5 ± 12.5%, *p* = 0.031^qq^, Code B 180° vs Code B 225° difference: 47.5 ± 13.77%, *p* = 0.12^rr^, *n* = 4 sessions). Increased complexity in the odor environment also caused a change in the path characteristics of the robot. For Code B, the path linearity decreased for several start angles (Fig. 4*E*; two-tailed *t* test port 1 135° with honeycomb vs port 1 135° no honeycomb difference: 0.17 ± 0.046, *p* = 0.0063^ss^, two-way ANOVA port 2, interaction between starting angle and plume complexity, *p* = 0.028, port 2 180° with honeycomb vs port 2 180° without honeycomb difference: 0.18 ± 0.051, *p* = 0.0068^tt^, *n* = 4 simulations).

When compared to *in silico* paths, Arduino-tested Code B trajectories are significantly more linear than *in silico*-tested Code B trajectories in both low-complexity and high-complexity environments (Extended Data Fig. 5-1*D*; two-tailed *t* test low-complexity robot Code B vs model Code B difference: 0.22 ± 0.071, *p* = 0.031^uu^, high-complexity robot Code B vs model Code B difference: 0.25 ± 0.071, *p* = 0.01^vv^). This discrepancy maybe be due to the wide range of starting angles tested for each odor port using *in silico* algorithms. Additionally, there is no significant difference between performance of Code B *in silico* and in the real flow chamber using the Arduino robot (Extended Data Fig. 5-1*A*, left; two-tailed *t* test low-complexity robot Code B vs model Code B difference: –11.07 ± 8.74%, *p* > 0.99^ww^, high-complexity robot Code B vs model Code B difference: –23.74 ± 8.74%, *p* = 0.07^xx^, *n* = 4 sessions). When model performance is determined selectively for the same start angles as tested on the robot, there is no significant difference between performance with low plume complexity between the robot and the model. Additionally, this subset of model data shows that the robot and the model show similar decreases in performance when the honeycomb is removed (Extended Data Fig. 5-1*A*, right; two-tailed *t* test low-complexity robot Code B vs model Code B difference: –34.16 ± 10.18%, *p* = 0.091, high-complexity robot Code B vs model Code B difference: –49.58 ± 9.48%, *p* < 0.0001, one-tailed *t* test robot Code B high vs low-complexity difference: –40.41 ± 11.01%, *p* = 0.028, one-tailed *t* test model Code B high vs low-complexity difference: –30.83 ± 11.72%, *p* < 0.0001, *n* = 4 conditions). Just as in the *in silico* model, the robot using Code B takes a significantly longer amount of time to reach the odor source on successful trials and has a significantly lower velocity when compared to mice (Extended Data Fig. 5-1*B*; two-tailed *t* test low-complexity mouse vs robot Code B time to target difference: –36.49 ± 3.63 s, *p* < 0.0001^yy^, high-complexity mouse vs Code B time to target difference: –41.55 ± 3.63 s, *p* < 0.0001^zz^, low-complexity mouse vs robot Code B velocity difference: 20.93 ± 1.44 cm/s, *p* < 0.0001^aaa^, high-complexity mouse vs robot Code B velocity difference: 29.06 ± 1.44 cm/s, *p* < 0.0001^bbb^, *n* = 4 mice, *n* = 4 sessions). Difference in trajectories between static and dynamic conditions can be observed in Figure 5*B*, Movies 12, 13, 14, 15, 16, 17, 18, and 19. Overall, our results show that when algorithms selected using *in silico* testing are implemented in a real flow chamber, our findings are comparable to those *in silico*. Additionally, just as in our *in silico* model, robot navigation shows a dramatic decrease in performance with increased odor plume complexity that is not observed in mouse behavior.

10.1523/ENEURO.0212-19.2019.f5-1 Extended Data Figure 5-1 Comparison of navigation parameters across modalities. ***A***, Performance (calculated as % success during a session) in mouse, robot using Code B, model using Code A, and model using Code B in low-complexity and high-complexity SOL (left). Performance of the robot and the model using Code B, both including only start angles tested on robot [90° and 135° for port 1 (corner port); 135°, 180°, and 225° for port 2 (center port)]. Each data point in this plot represents trials per combination of sensor distance (8 and 16 cm) and target odor port (port 1 and port 2 for robot, corner and center for model, right). ***B***, Same as ***A*** using time to target on successful trials. ***C***, Same as ***A*** using velocity. ***D***, Same as ***A*** using linearity score. All plots show mean ± SEM, *n* = 4 mice, *n* = 4 sessions for robot (one session per combination of sensor distance and sensor angle), *n* = 4 sessions for each model condition (one session for per combination of sensor distance and target odor port). Download Figure 5-1, TIF file.

Movie 12. Arduino robot navigation to airborne odor source with honeycomb using Code A with sensors at angle 0° and distance 8 cm. Odor source is middle port (port 2) and start angle is indicated in lower left corner (135°, 190°, and 225°). Video recorded at 30 Hz and played back at 90 Hz. 10.1523/ENEURO.0212-19.2019.video.12

Movie 13. Arduino robot navigation to airborne odor source with honeycomb using Code A with sensors at angle 45° and distance 8 cm. Odor source is middle port (port 2) and start angle is indicated in lower left corner (135°, 190°, and 225°). Video recorded at 30 Hz and played back at 90 Hz. 10.1523/ENEURO.0212-19.2019.video.13

Movie 14. Arduino robot navigation to airborne odor source with honeycomb using Code A with sensors at angle 0° and distance 16 cm. Odor source is middle port (port 2) and start angle is indicated in lower left corner (135°, 190°, and 225°). Video recorded at 30 Hz and played back at 90 Hz. 10.1523/ENEURO.0212-19.2019.video.14

Movie 15. Arduino robot navigation to airborne odor source with honeycomb using Code A with sensors at angle 45° and distance 16 cm. Odor source is middle port (port 2) and start angle is indicated in lower left corner (135°, 190°, and 225°). Video recorded at 30 Hz and played back at 90 Hz. 10.1523/ENEURO.0212-19.2019.video.15

Movie 16. Arduino robot navigation to airborne odor source using code B with sensors at angle 0° and distance 8 cm. Odor source is middle port (port 2), start angle is indicated in lower left corner (135°, 190°, and 225°), condition indicated in lower left corner (honeycomb and no honeycomb). Video recorded at 30 Hz and played back at 90 Hz. 10.1523/ENEURO.0212-19.2019.video.16

Movie 17. Arduino robot navigation to airborne odor source using code B with sensors at angle 45° and distance 8 cm. Odor source is middle port (port 2), start angle is indicated in lower left corner (135°, 190°, and 225°), condition indicated in lower left corner (honeycomb and no honeycomb). Video recorded at 30 Hz and played back at 90 Hz. 10.1523/ENEURO.0212-19.2019.video.17

Movie 18. Arduino robot navigation to airborne odor source using code B with sensors at angle 0° and distance 16 cm. Odor source is middle port (port 2), start angle is indicated in lower left corner (135°, 190°, and 225°), condition indicated in lower left corner (honeycomb and no honeycomb). Video recorded at 30 Hz and played back at 90 Hz. 10.1523/ENEURO.0212-19.2019.video.18

Movie 19. Arduino robot navigation to airborne odor source using code B with sensors at angle 45° and distance 16 cm. Odor source is middle port (port 2), start angle is indicated in lower left corner (135°, 190°, and 225°), condition indicated in lower left corner (honeycomb and no honeycomb). Video recorded at 30 Hz and played back at 90 Hz. 10.1523/ENEURO.0212-19.2019.video.19

## Discussion

Information from highly dynamic airborne odor plumes drives critical survival behaviors in animals. Variation in properties of these plumes can cause significant changes in odor-localization strategies (Mafra-Neto and Cardé, 1994; Keller and Weissburg, 2004). Here we compare the differences in odor navigation performance with increased plume complexity in mice, an *in silico*-simulated model, and an Arduino-based robot. We found that all three were able to successfully navigate to airborne odor sources. However, mouse performance remained robust when complexity within the plume was increased whereas *in silico* model and robot performance dropped. Thus, the simple binaral and temporal algorithms implemented in the model and robot are sufficient for successful navigation in a low-complexity environment, but these strategies are susceptible to declined performance when the plume becomes more chaotic. If not directly compared to mammalian odor-localization performance, these shortcomings in model performance may not have been effectively identified. With the goal of identifying minimalist biologically plausible rules that can capture animal navigation behavior, we highlight the importance of testing candidate algorithms in the same odor environment as behaving animals.

An increase in the chaotic nature of an odor environment has varying effects on odor source localization from species to species (Mafra-Neto and Cardé, 1994; Keller and Weissburg, 2004; Ferner and Weissburg, 2005; Jackson et al., 2007; Bhattacharyya and Bhalla, 2015). Our study shows that an increase in plume complexity does not affect successful odor localization in mice (Fig. 2*A*), a result that is in line with findings from Bhattacharyya and Bhalla (2015). Additionally, we show that an increase in plume complexity causes a significant decrease in time to the odor source on successful trials and an increase in speed throughout the trial (Fig. 2*E*,*G*). Speed and sniff rate are positively correlated and this correlation peaks at a lag where velocity precedes sniff frequency (Coronas-Samano et al., 2016; Jones and Urban, 2018). We speculate that an animal’s increase in speed during odor tracking when the odor environment becomes more chaotic, as measured by the increase in SD of concentration, may drive sniffing at higher frequencies (although not directly measured) to detect fluctuations in the odor plume. This would suggest that to remain equally successful at odor localization with increased plume complexity, mice may have to implement a different innate navigation strategy. To address this hypothesis, further work needs to be done to explore changes in sampling behavior with changes in odor plume properties. Our finding of a shift to faster navigation in more chaotic environment in mice is contrary to the decreased navigational speed with increased plume complexity observed by Bhattacharyya and Bhalla (2015) in rats. The discrepancy between these two findings may be due to task design. We specifically designed our odor navigation task to require mice to take direct paths to odor sources, instead of serially checking all possible odor ports, unlike previous studies (Bhattacharyya and Bhalla, 2015; Gire et al., 2016). We did so by terminating trials after animals reached any of the three ports. The nature of the odor-localization task design could be critical to the observation of different navigational strategies.

Animals, both vertebrates and invertebrates alike, often implement a “zig-zagging” strategy while navigating odor environments, often to detect the boundary of odor presence (Vickers, 2000; Grasso, 2001; Porter et al., 2007; Khan et al., 2012; Catania, 2013). However, recent studies characterizing rodent navigation behavior within odor plumes show a lack of casting while localizing airborne odors (Bhattacharyya and Bhalla, 2015; Gire et al., 2016). In line with these studies, we find that mice display paths with little curvature while navigating an airborne odor plume, on average turning less than a full rotation on a given trial, although their navigation arena in our task was nearly 1 m^2^. However, interestingly, and not contradictory to previous observations, we find that mice do display a significant amount of lateral nose movement during navigation, predominantly early on in odor-tracking. As found in previous studies showing casting behavior in mammals while tracking odor trails, this early lateral nose movement, although speculative, may be used to detect the boundary of the odor plume (Fig. 2*H*,*I*).

Here, we explored the odor navigation performance of two minimal algorithms: Code A relied solely on binaral comparisons and movement in the direction of higher concentration, while Code B made temporal comparisons between consecutive time points to determine direction of concentration gradient before defaulting to Code A. Using our *in silico* model, we found that Code A performed better at a larger sensor separation distance than Code B and Code B performed better at a smaller sensor separation distance than Code A (Fig. 3*D*). With a smaller sensor separation distance, the concentration readings at both of the sensors were closer in value than those when the sensors were at a larger separation distance (Extended Data Figs. 3-1-3-5). Code B relies on a comparison between an average of the two sensor readings at sequential time points. These comparisons will be more accurately representative of true odor gradient increases when based on more correlated sensor readings. Further, when the sensors are closer together, they are also closer to the midline of the robot, and most related to the robot’s trajectory. Thus, this may explain the lower success rate of Code B in comparison to Code A at larger sensor separation distances. However, at a shorter sensor distance, when sensors will have more similar readings, the additional temporal strategy shows improved success. Additionally, at an 8-cm separation distance, Code B showed a spatial periodicity in performance and linearity where the two parameters cycled every 30° of starting angles (Extended Data Fig. 3-6). The model makes turns at increments of 30° and an optimal performance is observed when the model is able to achieve an angle of 180° (directly facing the odor port) by turning. The complexities of our algorithms are limited as the goal of the present study was to address how well minimal, but biologically plausible, algorithms can perform odor navigation in a real plume and how it deviates from mammalian behavior. Thus, future studies should explore how to best optimize turning behavior to maximize successful start angles, possibly trading off the coarseness of turning (and step size and step frequency in general) for the speed of path adjustment. In addition, further work is needed to probe algorithm dependence on parameter adjustment, such as implementation of corrective movement and altering sampling speed. The ability to collect enough simulations to make these comparisons highlights the benefit of testing navigational algorithms *in silico*.

When we directly compared the performance and behavior of the mice to that of the *in silico* model and robot in the same odor environment, we found that mouse odor-localization success was more robust to changes in plume complexity than that of the model or robot. Mice are able to modulate their sampling behavior by altering sniff frequency, thus sampling is dynamic throughout the odor navigation process (Verhagen et al., 2007; Wesson et al., 2008, 2009; Khan et al., 2012; Bhattacharyya and Bhalla, 2015; Jones and Urban, 2018; Jordan et al., 2018; Shusterman et al., 2018). Additionally, mice are able to modulate their running speed, as our data show an increase in speed during the middle of the trajectory and slower speeds at the beginning and end (Fig. 2*E*,*G*). As suggested previously, this modulation of speed may be beneficial for controlling optimal sampling frequency which may vary based on position in the odor plume. Contrary to the mouse, the model and robot algorithms we tested do not allow for sampling modulation. Due to the complex and highly dynamic structure of odor plumes, a fixed sampling frequency may result in a limited perception of odor presentation at a given point within the plume. The ability to modulate behavior in real time during navigation is likely an important factor contributing to consistent performance with changes in odor plume properties. In addition, although not measured in our study, whisking behavior drives localization of wind direction in mice (Yu et al., 2016). Wind direction is critical for odor source localization in insects. Although the role of anemotaxis in odor localization in rodents is understudied, whisking is correlated with sniffing behavior (Shusterman et al., 2011; Moore et al., 2013; Kleinfeld et al., 2014; Kurnikova et al., 2017), and thus may be highly modulated during odor navigation. Further work is needed to understand the role of whisking behavior in odor localization and in tandem, how adding anemometry to model and robot algorithms affects navigation performance.

Our study reveals the benefit of comparing different systems (i.e., animals, robots, and models) on odor-localization behavior in the same environment. We were able to address the question of to what degree minimal spatial and temporal algorithms can account for mouse navigation behavior. Our data show that simple spatial and temporal algorithms can perform as well as mice in a low-complexity odor environment, but poorer when odor plumes become more dynamic. This suggests that mice implement more complex strategies than our minimal equivalent algorithms. Thus, for robust mouse-like behavior, our minimal algorithms driving models or robots must be made more complex. Additionally, as mentioned previously, animals may display different navigation behaviors based on the behavioral arena and task structure. By testing all systems in the same environment and on the same task, we were able to reveal differences that would not have been uncovered otherwise. Future studies need to focus on testing simulations in tandem with behaving animals in a naturalistic, chaotic odor environment to best understand how odor-localization algorithms perform compared to animal behavior. Through such studies, algorithms that incorporate dynamic sampling and other sensory measurements in addition to olfaction may show behavior equally robust to that of animals. Such studies will serve to complement more normative non-mechanistic models such as infotaxis (Vergassola et al., 2007; Yang et al., 2018), which, while providing optimal decisions on whether to explore versus exploit in a “greedy” fashion, do not address questions about biological plausibility of navigation algorithms.

## Synthesis

Reviewing Editor: Anna Menini, SISSA (International School of Advances Studies) Decisions are customarily a result of the Reviewing Editor and the peer reviewers coming together and discussing their recommendations until a consensus is reached. When revisions are invited, a fact-based synthesis statement explaining their decision and outlining what is needed to prepare a revision will be listed below. The following reviewer(s) agreed to reveal their identity: Michael Schmuker.

The manuscript describes odor navigation experiments with mice and robots. It is analysed how the performance of mice and robots change when increasing the amount of turbulence, under the premise that mice perform better when turbulence increases. Robots are equipped with “bi-naral” sensors and some measures have been taken to improve the sensing speed. The main observation appears to be that mouse odor navigation remains stable when turbulence increases, whereas robots fail to navigate under those conditions.

Both reviewers have several specific suggestions for improving the manuscript. Please carefully address the specific concerns listed below.

1. A major criticism of this work is that it is unclear what novel insight is added. The odor navigation algorithms are very simplistic and it is therefore not at all surprising that they do not perform well. In general it is unclear how the principles underlying the algorithmic design correspond to mouse odor navigation strategies, or if this was a design principle at all. While the algorithms integrate bi-naral information, it is unclear whether this integration is somehow related to how mice perform that task. Established odor navigation algorithms like infotaxis and casting/surging are not even discussed in the manuscript, which is a glaring omission in the light of the vast body of literature available.

2. The manuscript is exceptionally hard to read. The author guidelines probably caused the frequent and lengthy quotation of statistical tests in the main text. But isn't there a way to include this information that is less disruptive to the reading flow? Part of the information given in the text is also redundant to the information given in the table.

The manuscript would greatly benefit from splitting paragraphs into smaller units for the sake of readability.

If the authors had followed the guidelines and used double spacing this would probably have improved things already.

3. A back of the envelope calculation shows that the the Reynolds number for the flow ranges from 15 to 125 depending on the choice of length scale. This falls in the range of weak to transitional turbulence which does not have the “highly dynamic” and “filamentous” structure that is invoked by the authors.

4. The authors state that “to increase turbulence” they removed the inlet honeycomb. However, no direct airflow measurement is provided to validate this claim. The extended data in Fig 1.1 show that there is some measurable change in the odor concentration, but it is well known that even non turbulent but chaotic flows can produce highly irregular concentration patterns with strong sensitivity on external parameters. The claim is therefore not supported by available experimental evidence.

5. The rationale for an increased search speed by mice in the flow without honeycomb is speculative: there is no direct evidence of an increase in the frequencies of the odor signal that would motivate higher sniff frequencies (also not measured) and therefore higher speeds.

6. The comparison with in silico/robotic search is of limited interest as it only considers two simple reactive algorithms and there is no extensive analysis of the parameter dependence (update rate, duration of turns and backups, equilibration time...) It is difficult to assess if the reduced performance is to be attributed to the lack of adaptive sensing. Also, it would not be difficult to implement the in silico version of sniffing.

Minor aspects:

l 312 - Make, model, vendor of the Arduino robot needs to be stated.

l 341ff - unclear how a hole behind the sensor increases airflow - is the sensor's back open?

Figure 4: fonts are too small. Also there is very little discussion of the figure in the text. I'm not sure what insight it adds.

Fig. 5-1A Too small and resolution insufficient to resolve relevant detail of the sensing apparatus.

Fig. 5-1B Add a description on how these data were acquired.

Fig. 6: Arena coordinates are different in mouse and robot experiments but this is not explained. Also, why is code A not shown?

l741-744 - the causal link between these two sentences is very weak.
