# Supplementary figures and images for: A Comparison between Mouse, In Silico, and Robot Odor Plume Navigation Reveals Advantages of Mouse Odor Tracking
Source: eNeuro. 2020 Jan 31;7(1):ENEURO.0212-19.2019. doi: 10.1523/ENEURO.0212-19.2019 (PMC7004486; doi:10.1523/ENEURO.0212-19.2019)

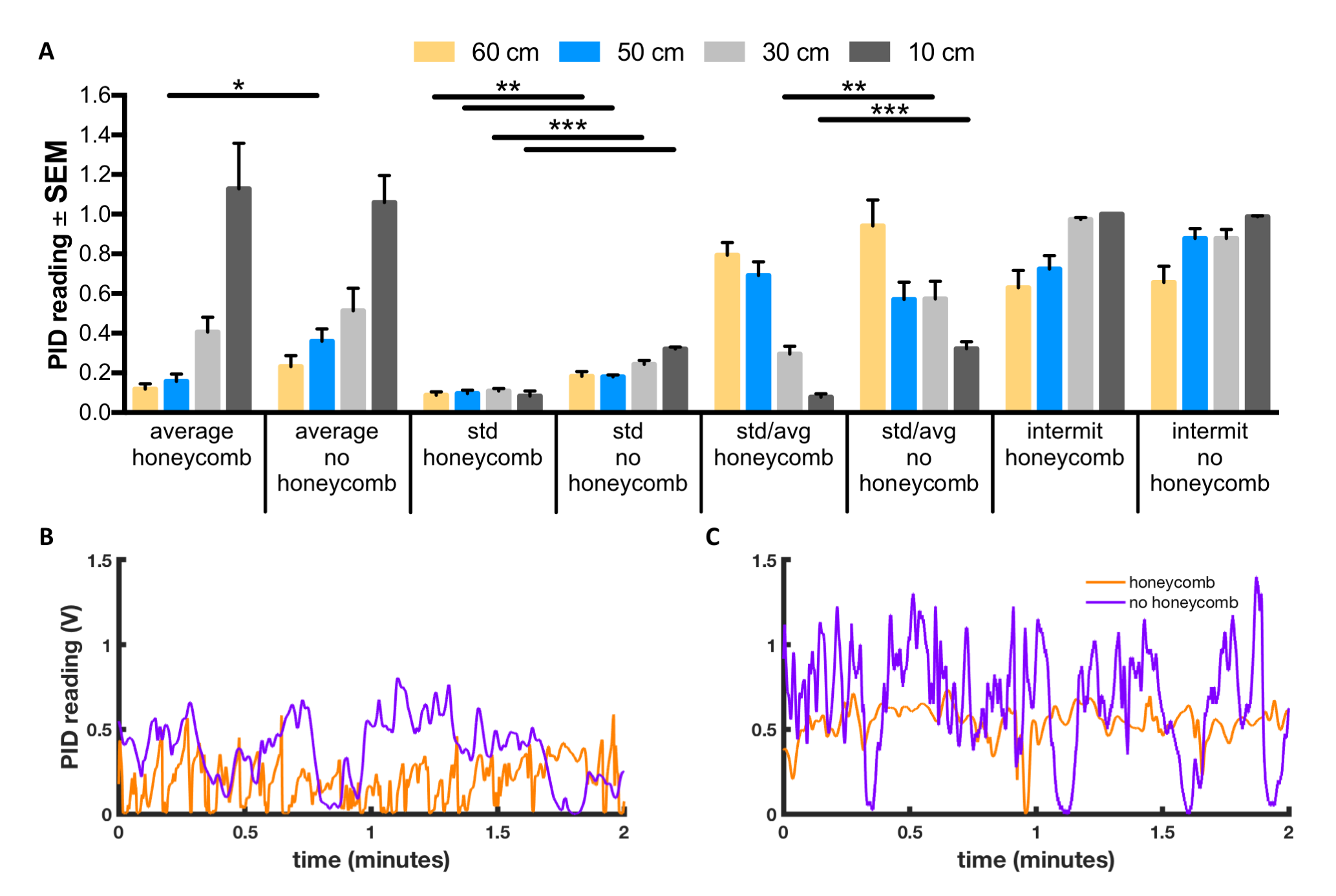

Supplement: Extended Data Figure 1-1 — Odor plume within the SOL with and without honeycomb. A, Odor plume properties within the SOL with and without the inlet air laminarization honeycomb at 10, 30, 50, and 60 cm downstream from odor tube. The average miniPID reading at 50 cm from the odor tube is greater without the honeycomb when compared to with the honeycomb (one-tailed t test with correction for multiple comparisons, average with honeycomb 0.16 ± 0.04, average no honeycomb: 0.36 ± 0.06, p = 0.040). The SD of the PID reading at all distances from the outlet is greater without the honeycomb than with the honeycomb (one-tailed t test with correction for multiple comparisons, 60-cm std with honeycomb: 0.09 ± 0.02, 60-cm std no honeycomb: 0.18 ± 0.02, p = 0.014; 50-cm std with honeycomb: 0.10 ± 0.02, 50-cm std no honeycomb: 0.18 ± 0.01, p = 0.004; 30-cm std with honeycomb: 0.11 ± 0.01, 30-cm no honeycomb: 0.25 ± 0.02, p < 0.0001; 10-cm with honeycomb: 0.08 ± 0.03, 10-cm no honeycomb: 0.32 ± 0.01, p < 0.0001). The std/average is greater without the honeycomb than with the honeycomb at 30 and 10 cm from the odor tube (one-tailed t test with correction for multiple comparisons, 30-cm std/average with honeycomb: 0.29 ± 0.04, 30-cm std/average no honeycomb: 0.57 ± 0.09, p = 0.033; 10-cm std/average with honeycomb: 0.08 ± 0.02, 10-cm std/average no honeycomb: 0.32 ± 0.04, p < 0.0001). B, Example PID readings for honeycomb and no honeycomb conditions from 2-min sample at 60 cm from the source. C, Example PID readings for honeycomb and no honeycomb conditions from 2-min sample at 30 cm from the source. Download Figure 1-1, TIF file. [file sup_enu-eN-NWR-0212-19-s01.tif]

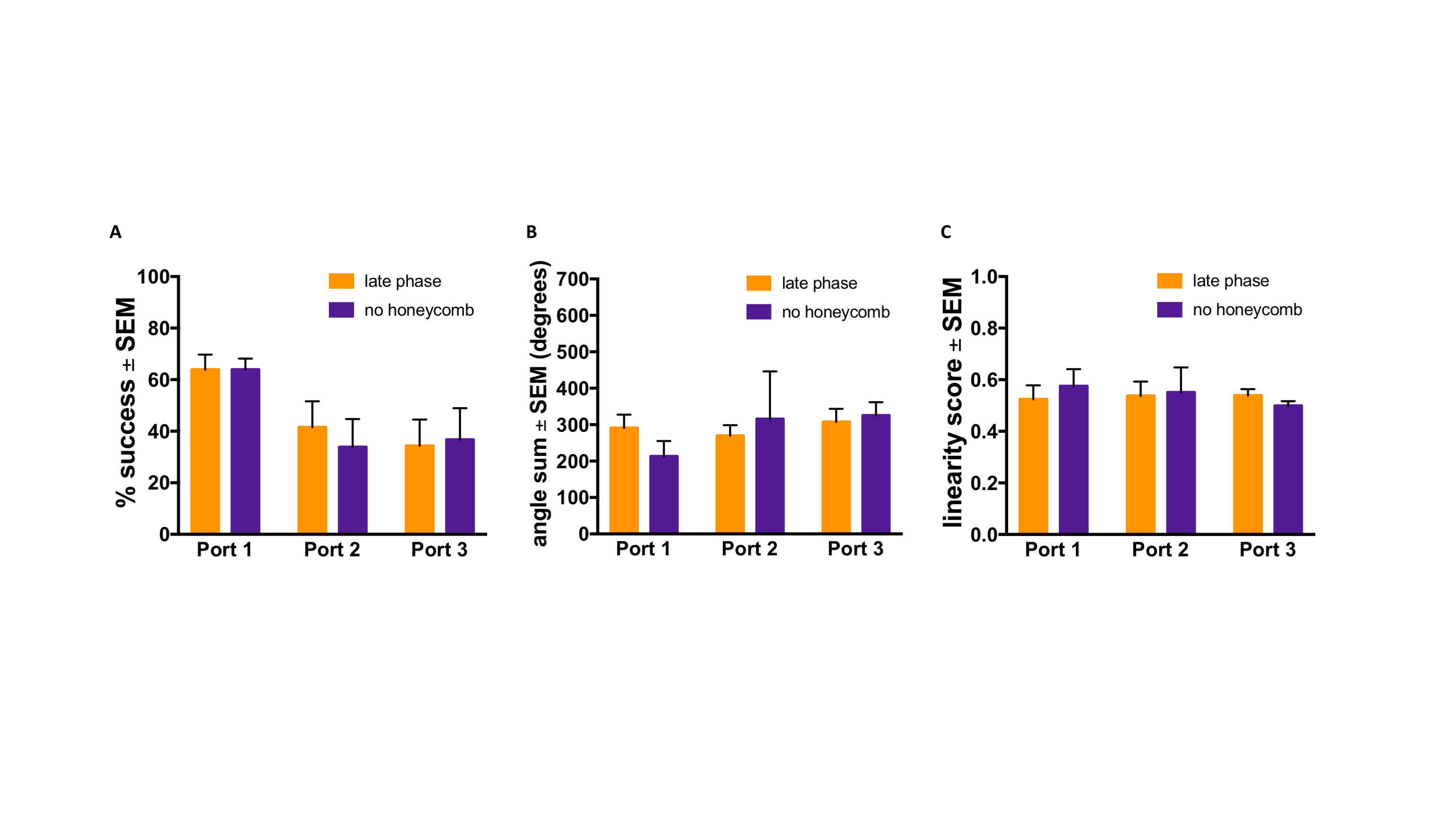

Supplement: Extended Data Figure 2-1 — Mice show consistent performance and turning behavior across both low-complexity and high-complexity odor environments. A, % success of mouse navigation at each target odor port in the late phase and no honeycomb conditions. B, Same as A, for total angle sum. C, Same as A, for linearity score. All plots show mean ± SEM, n = 4 mice. Download Figure 2-1, TIF file. [file sup_enu-eN-NWR-0212-19-s02.tif]

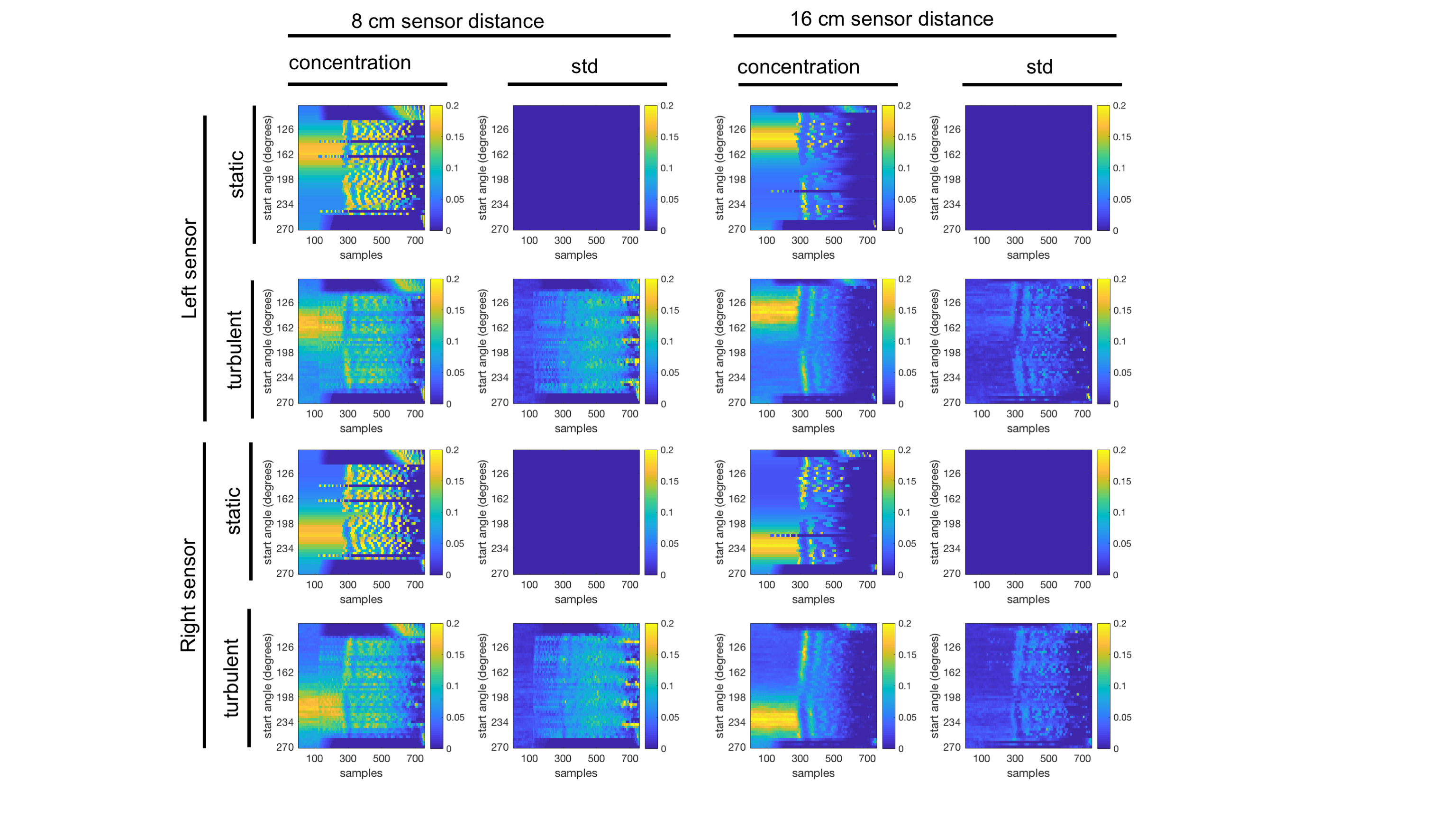

Supplement: Extended Data Figure 3-1 — Instantaneous concentration for in silico algorithm Code A at center start position over trajectories resampled to 755 frames. Each trajectory was resampled to 755 frames (the maximum amount of time the model was allotted) and averaged across starting angle (y-axis). Twenty simulations per starting angle were tested. Concentration shown with color scale. For first ∼275 samples, the model is stationary due to collecting baseline data, thus the odor concentration does shows little variation during this sampling period. Data are grouped by left and right sensor reading, tested odor plume (static or dynamic), and sensor separation distance (8 and 16 cm). For each condition, the average concentration at each starting angle is plotted as well as the SD of concentration on these trajectories. Download Figure 3-1, TIF file. [file sup_enu-eN-NWR-0212-19-s03.tif]

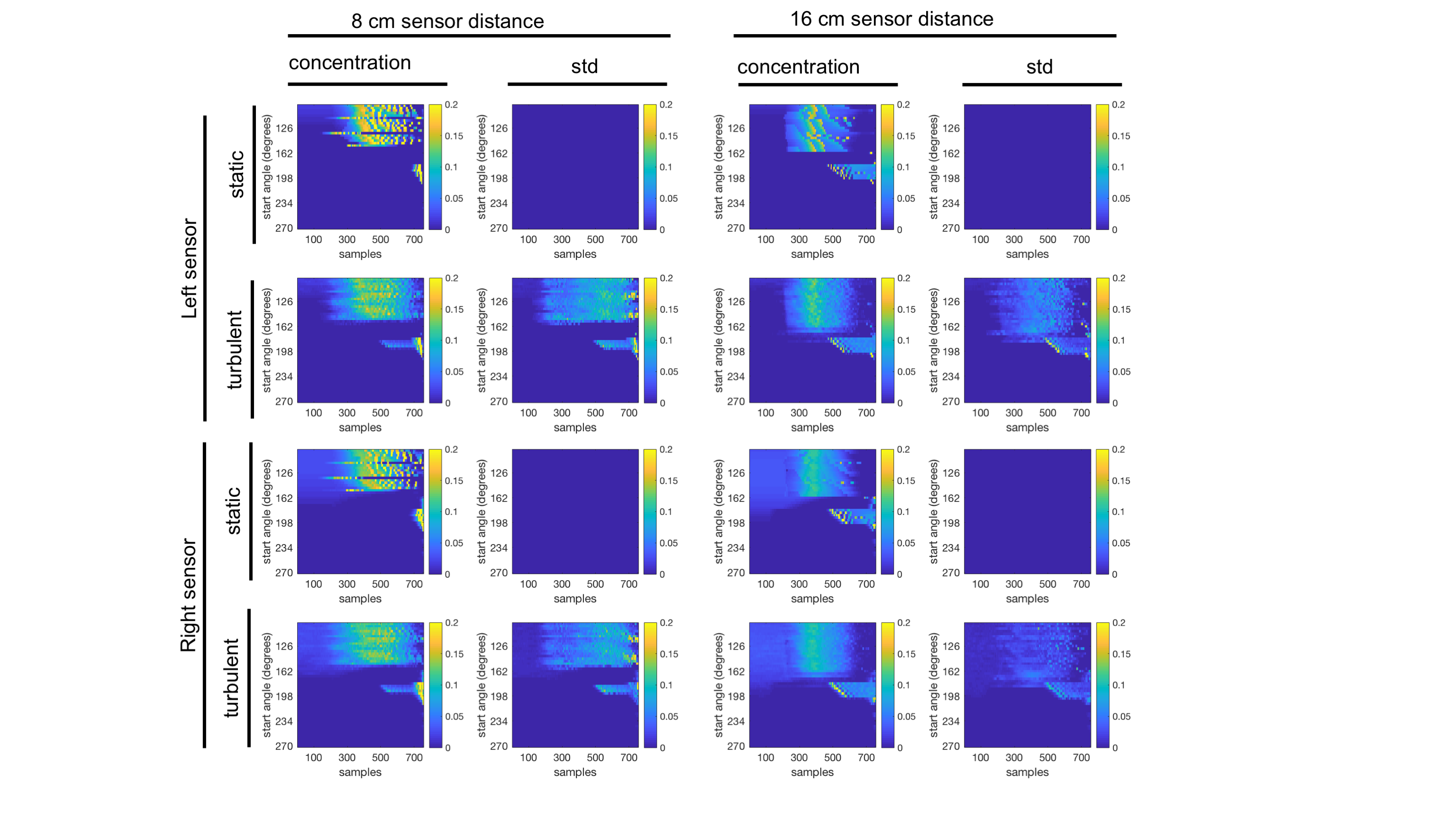

Supplement: Extended Data Figure 3-2 — Instantaneous concentration for in silico algorithm Code A at corner start position over trajectories resampled to 755 frames. Each trajectory was resampled to 755 frames (the maximum amount of time the model was allotted) and averaged across starting angle (y-axis). Twenty simulations per starting angle were tested. Concentration shown with color scale. For first ∼275 samples, the model is stationary due to collecting baseline data, thus the odor concentration does shows little variation during this sampling period. Data are grouped by left and right sensor reading, tested odor plume (static or dynamic), and sensor separation distance (8 and 16 cm). For each condition, the average concentration at each starting angle is plotted as well as the SD of concentration on these trajectories. Download Figure 3-2, TIF file. [file sup_enu-eN-NWR-0212-19-s04.tif]

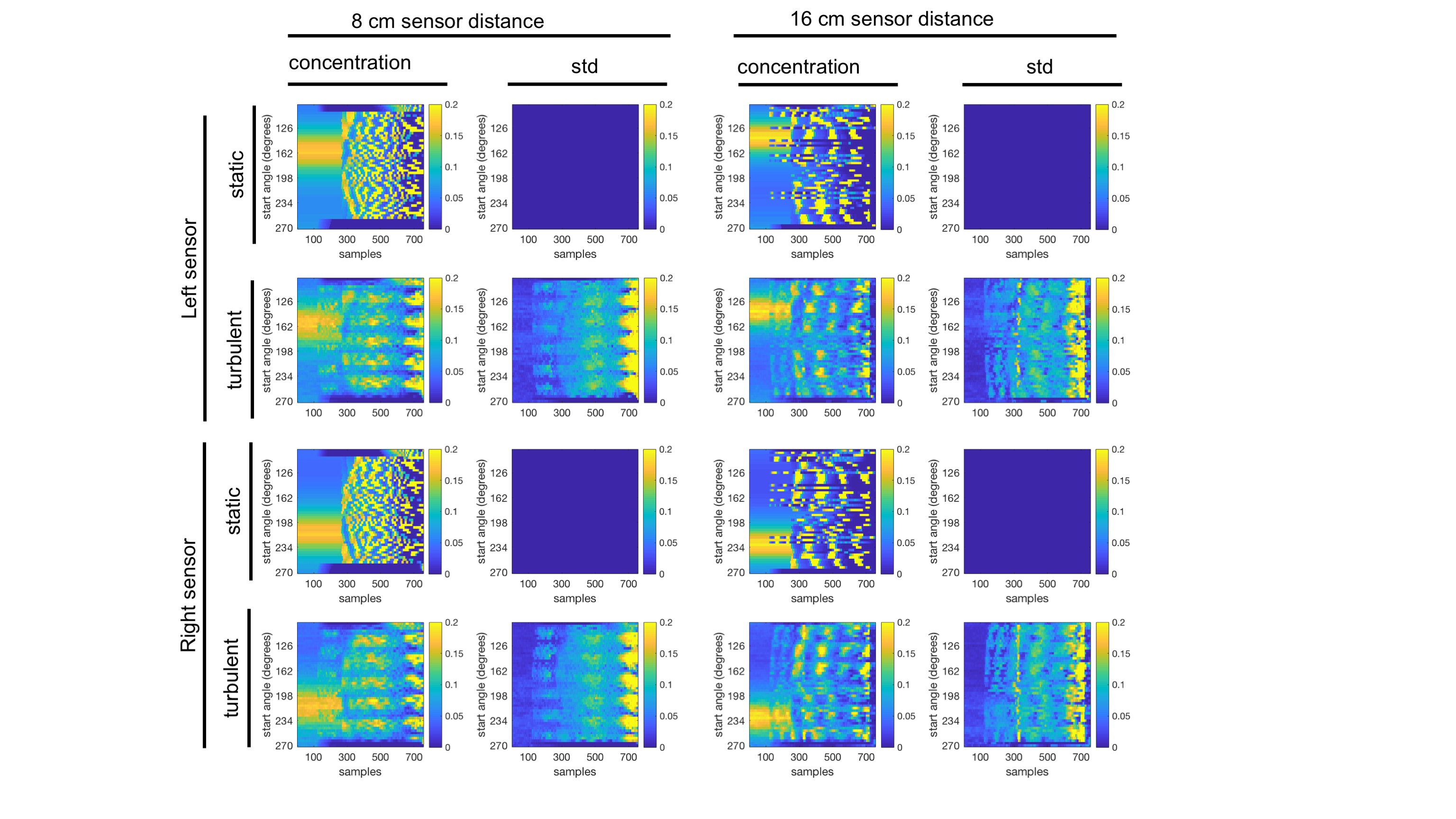

Supplement: Extended Data Figure 3-3 — Instantaneous concentration for in silico algorithm Code B at center start position over trajectories resampled to 755 frames. Each trajectory was resampled to 755 frames (the maximum amount of time the model was allotted) and averaged across starting angle (y-axis). Twenty simulations per starting angle were tested. Concentration shown with color scale. For first ∼275 samples, the model is stationary due to collecting baseline data, thus the odor concentration does shows little variation during this sampling period. Data are grouped by left and right sensor reading, tested odor plume (static or dynamic), and sensor separation distance (8 and 16 cm). For each condition, the average concentration at each starting angle is plotted as well as the SD of concentration on these trajectories. Download Figure 3-3, TIF file. [file sup_enu-eN-NWR-0212-19-s05.tif]

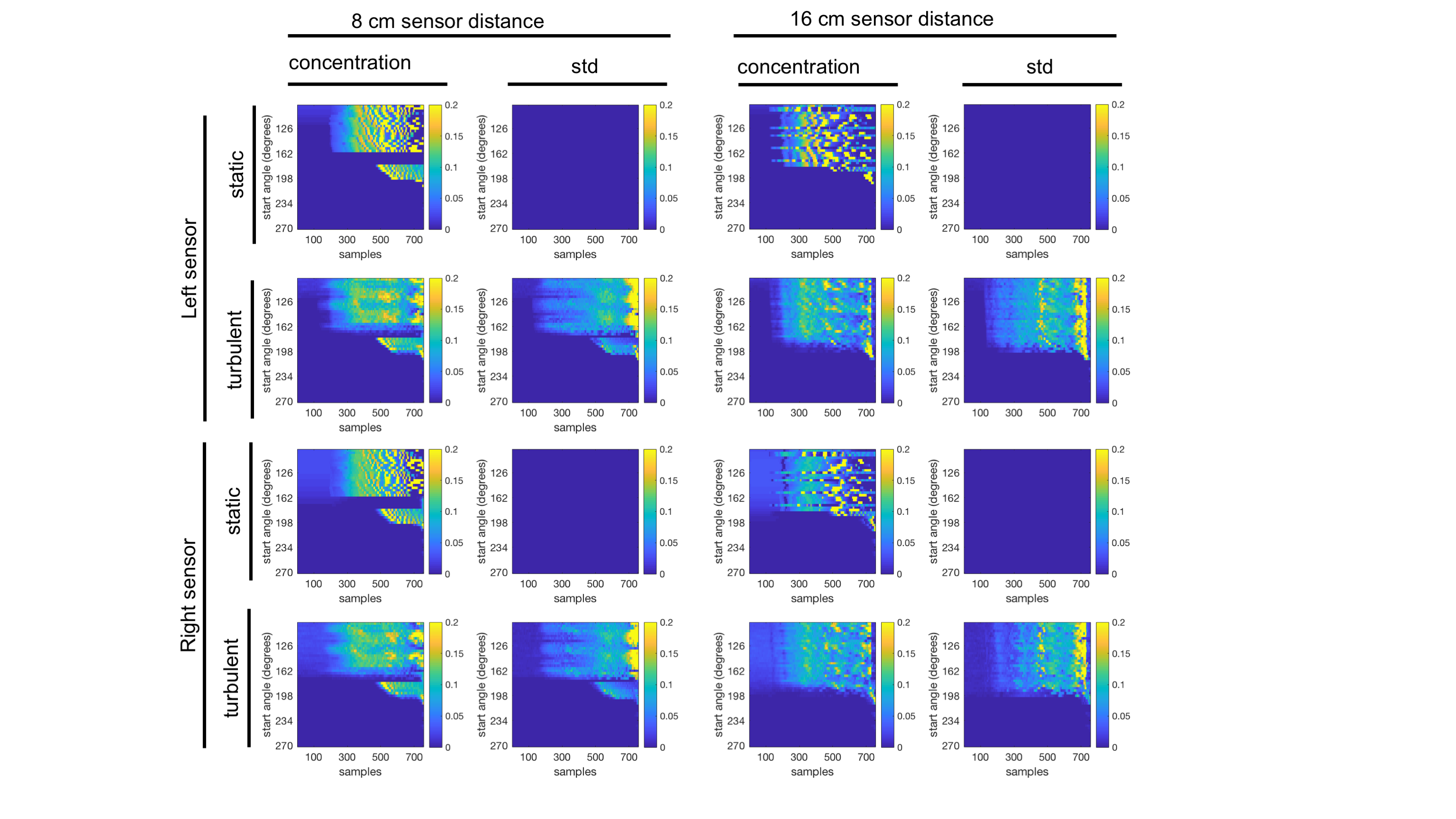

Supplement: Extended Data Figure 3-4 — Instantaneous concentration for in silico algorithm Code B at corner start position over trajectories resampled to 755 frames. Each trajectory was resampled to 755 frames (the maximum amount of time the model was allotted) and averaged across starting angle (y-axis). Twenty simulations per starting angle were tested. Concentration shown with color scale. For first ∼275 samples, the model is stationary due to collecting baseline data, thus the odor concentration does shows little variation during this sampling period. Data are grouped by left and right sensor reading, tested odor plume (static or dynamic), and sensor separation distance (8 and 16 cm). For each condition, the average concentration at each starting angle is plotted as well as the SD of concentration on these trajectories. Download Figure 3-4, TIF file. [file sup_enu-eN-NWR-0212-19-s06.tif]

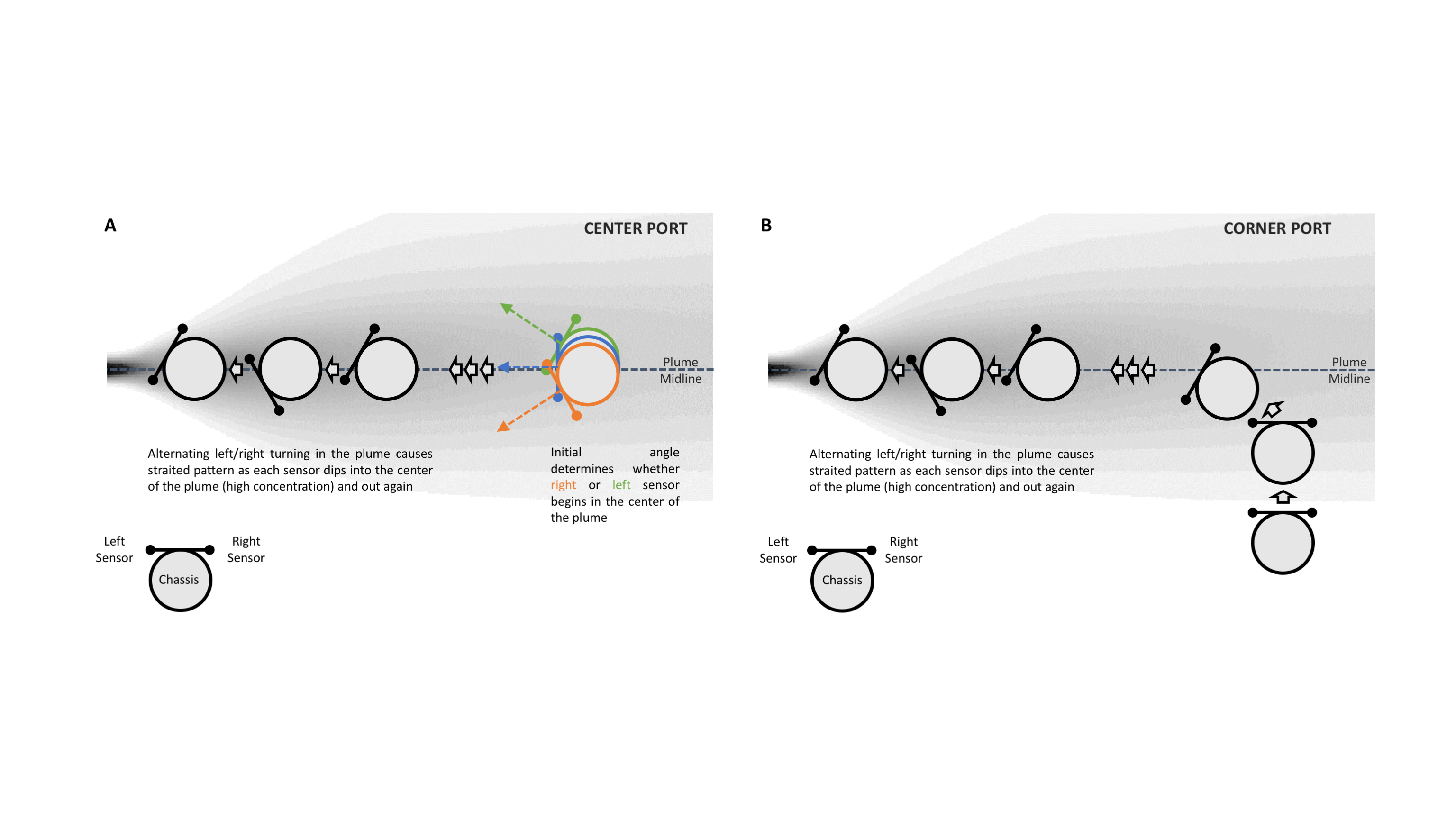

Supplement: Extended Data Figure 3-5 — Schematics of model trajectories through odor plume. A, Example model trajectory through center odor plume. Striated patterning seen in Extended Data Figures 3-1, Figures 3-4 is due to robot rotating, causing sensors to rotate in and out of the odor plume. Striated patterning is more obvious at 16-cm sensor separation distance due to sensors being wider apart and therefore detecting odor environments with greater concentration differences. Additionally, striated patterning is less obvious in the dynamic plume because the plume is dynamic and the paths are not deterministic, so averages across trials will show a smoother gradient of concentration over trial time. B, Example model trajectory through corner odor plume. Model begins out of the odor plume, and therefore, the first several frames in Extended Data Figure 3-3, Figure 3-4 show a very low concentration. Again, striated patterning is more obvious at 16-cm sensor separation distance and less obvious in the dynamic plume condition. Download Figure 3-5, TIF file. [file sup_enu-eN-NWR-0212-19-s07.tif]

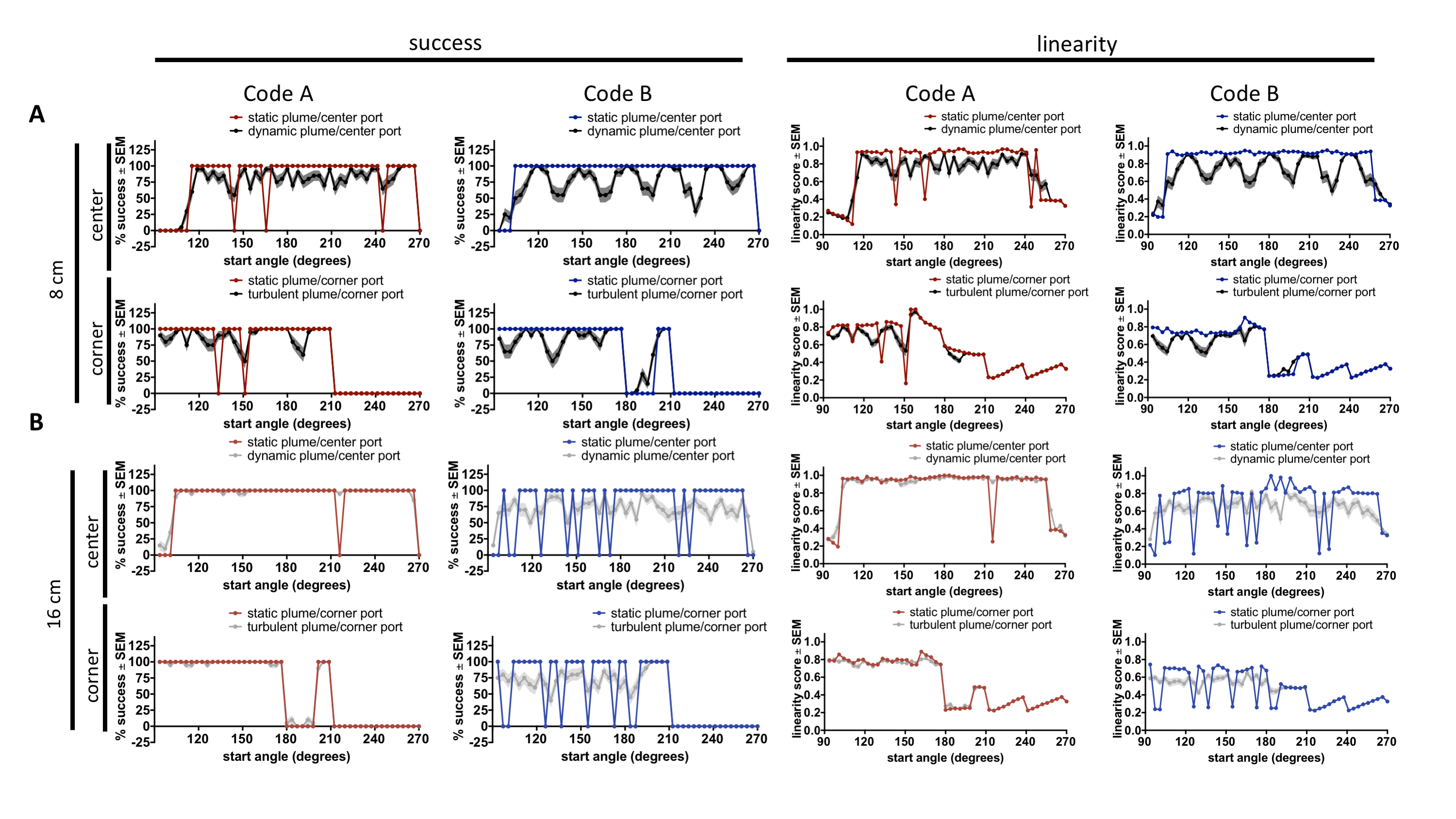

Supplement: Extended Data Figure 3-6 — Navigation performance and trajectory linearity across start angles. A, % success (mean performance of one simulation with all start angles tested) and linearity score with static and dynamic plume using binaral model (Code A) and temporal-based binaral model (Code B) across starting angles with a sensor separation distance of 8 cm. Graphs are grouped target port location (either center port or corner port). Plots show mean % success ± SEM or mean linearity score ± SEM; n = 20 simulations, Code A shown in red, Code B shown in blue. B, Same as A, for a sensor separation distance of 16 cm. Download Figure 3-6, TIF file. [file sup_enu-eN-NWR-0212-19-s08.tif]

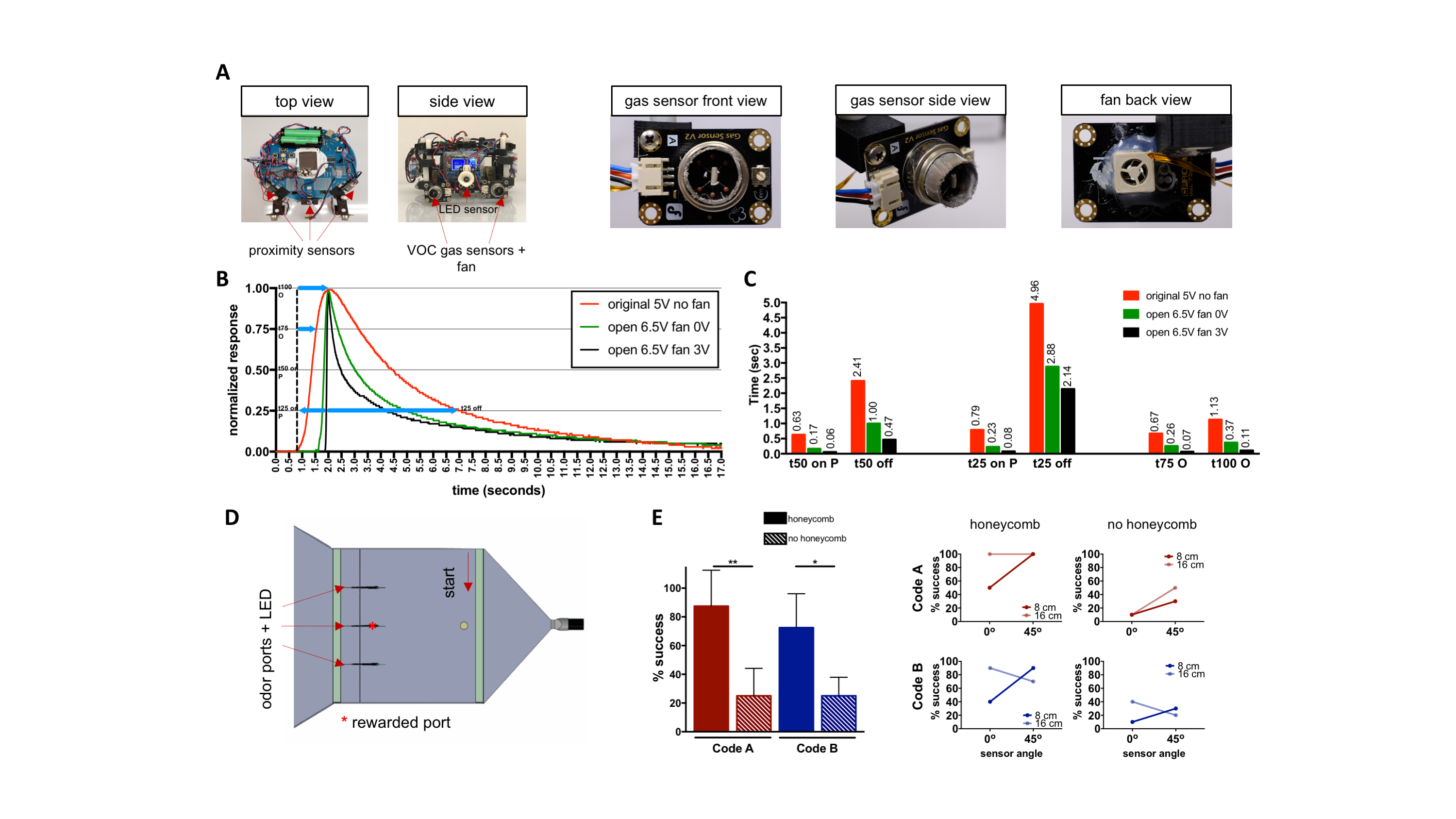

Supplement: Extended Data Figure 4-1 — Increased odor plume complexity impairs Arduino-based robot navigation from alternate starting position. A, Top and side view of robot with three proximity, two VOC gas sensors with fans, and an LED sensor. B, C, Normalized odor concentration reading after brief ethanol exposure over time with an original sensor powered at 5 V (1.25 W per sensor), a modified sensor with fan at 6.5 V (2 W) without driving the fan, and a modified sensor with fan at 6.5 V and driving the fan using 3 V (0.15 W). t50 on P: rise time from t50 (time at 50% of peak amplitude) to tp (peak amplitude). t50 off: decay time from to tp to t50. t25 on P: rise time from t25 (25% of peak amplitude) to tp (peak amplitude). t25 off: decay time from to tp to t25. t75 on O: rise time from response onset (2% of peak amplitude) to t75 (75% of peak amplitude). t100 on O: rise time from response onset (2% of peak amplitude) to t100 (peak amplitude). D, Robot odor navigation flow chamber. Red arrow labeled “start” indicates the alternate starting position and the red asterisk indicates the active odor port. E, Performance (average % successful trials over 8 and 16 cm and 0° and 45° gas sensor distance and angles, respectively) across codes with and without honeycomb. Plot shows mean % success ± SEM, n = 4 sessions (left). Performance based on gas sensor distance (8 and 16 cm) and angle (0° and 45°) for the honeycomb and no honeycomb conditions (right). Download Figure 4-1, TIF file. [file sup_enu-eN-NWR-0212-19-s09.tif]

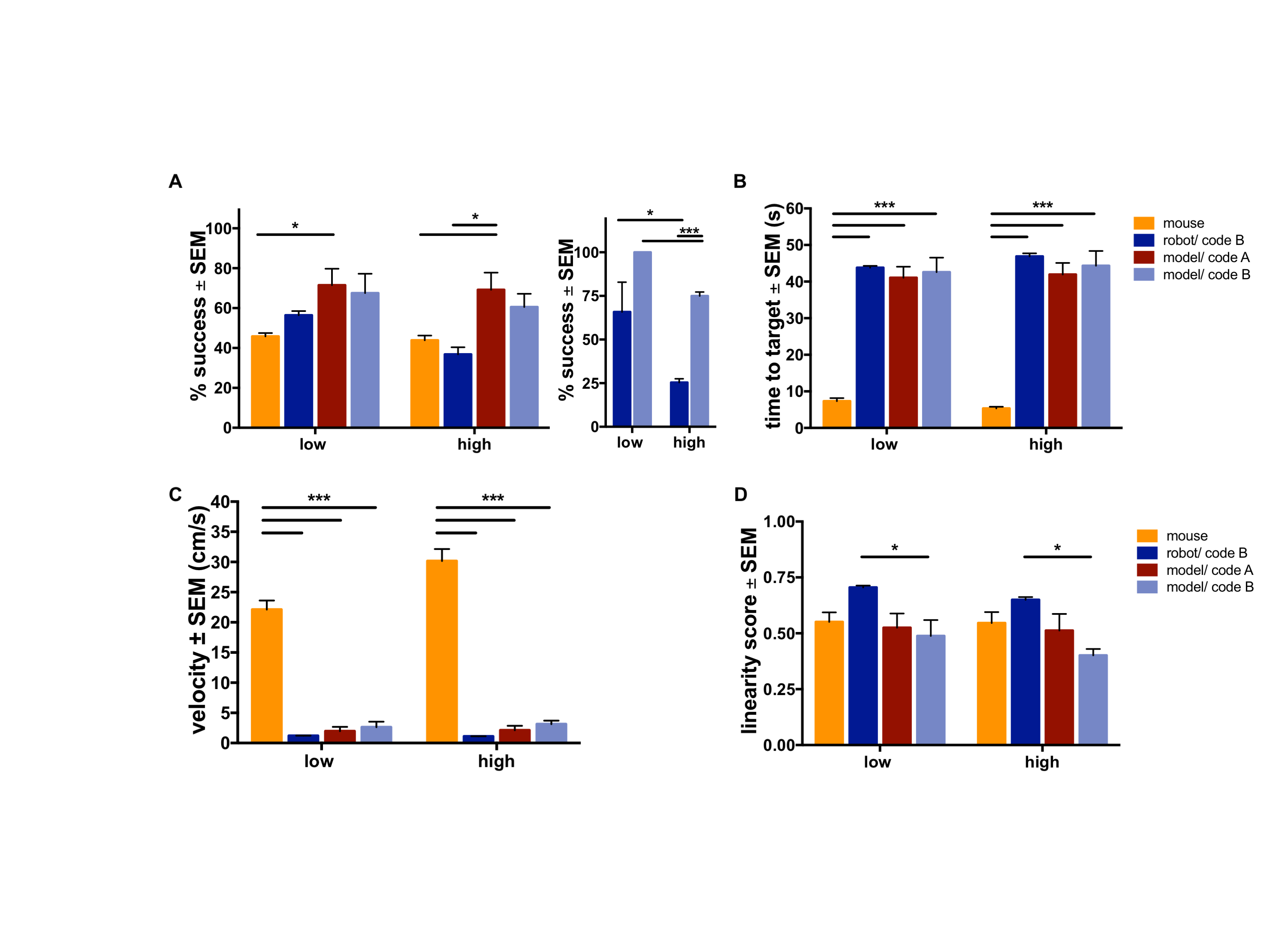

Supplement: Extended Data Figure 5-1 — Comparison of navigation parameters across modalities. A, Performance (calculated as % success during a session) in mouse, robot using Code B, model using Code A, and model using Code B in low-complexity and high-complexity SOL (left). Performance of the robot and the model using Code B, both including only start angles tested on robot [90° and 135° for port 1 (corner port); 135°, 180°, and 225° for port 2 (center port)]. Each data point in this plot represents trials per combination of sensor distance (8 and 16 cm) and target odor port (port 1 and port 2 for robot, corner and center for model, right). B, Same as A using time to target on successful trials. C, Same as A using velocity. D, Same as A using linearity score. All plots show mean ± SEM, n = 4 mice, n = 4 sessions for robot (one session per combination of sensor distance and sensor angle), n = 4 sessions for each model condition (one session for per combination of sensor distance and target odor port). Download Figure 5-1, TIF file. [file sup_enu-eN-NWR-0212-19-s10.tif]
